# Birth, expansion, and death of *VCY*-containing palindromes on the human Y chromosome

**DOI:** 10.1186/s13059-019-1816-y

**Published:** 2019-10-14

**Authors:** Wentao Shi, Andrea Massaia, Sandra Louzada, Juliet Handsaker, William Chow, Shane McCarthy, Joanna Collins, Pille Hallast, Kerstin Howe, Deanna M. Church, Fengtang Yang, Yali Xue, Chris Tyler-Smith

**Affiliations:** 1 The Wellcome Sanger Institute, Hinxton, Cambridgeshire, CB10 1SA UK; 2 Department of Genetics, School of Basic Medical Sciences, Tianjin Medical University, Tianjin, 300070 China; 3 Present address: National Heart and Lung Institute, Imperial College London, London, SW7 2AZ UK; 4 Present address: Department of Genetics, University of Cambridge, Cambridge, CB2 3EH UK; 5 Institute of Biomedicine and Translational Medicine, University of Tartu, 51011 Tartu, Estonia; 6 10x Genomics, 7068 Koll Center Parkway, Suite 401, Pleasanton, CA 94566 USA; 7 Present address: Inscripta Inc., 5500 Central Avenue #220, Boulder, CO 80301 USA

**Keywords:** Structural variation, Copy number variation, Inverted repeat, Sex chromosome, Variable Charge Y gene

## Abstract

**Background:**

Large palindromes (inverted repeats) make up substantial proportions of mammalian sex chromosomes, often contain genes, and have high rates of structural variation arising via ectopic recombination. As a result, they underlie many genomic disorders. Maintenance of the palindromic structure by gene conversion between the arms has been documented, but over longer time periods, palindromes are remarkably labile. Mechanisms of origin and loss of palindromes have, however, received little attention.

**Results:**

Here, we use fiber-FISH, 10x Genomics Linked-Read sequencing, and breakpoint PCR sequencing to characterize the structural variation of the P8 palindrome on the human Y chromosome, which contains two copies of the *VCY* (*Variable Charge Y*) gene. We find a deletion of almost an entire arm of the palindrome, leading to death of the palindrome, a size increase by recruitment of adjacent sequence, and other complex changes including the formation of an entire new palindrome nearby. Together, these changes are found in ~ 1% of men, and we can assign likely molecular mechanisms to these mutational events. As a result, healthy men can have 1–4 copies of *VCY*.

**Conclusions:**

Gross changes, especially duplications, in palindrome structure can be relatively frequent and facilitate the evolution of sex chromosomes in humans, and potentially also in other mammalian species.

## Background

Palindromes are inverted-repeat structures that form major parts of the sex chromosomes of humans (chrX, 2%; chrY, 30% of the euchromatin) and other species [1–7], but are less abundant on autosomes [8]. Some aspects of their genome biology are well-understood. We know that they show high sequence identity between the arms which is maintained by ongoing intrachromosomal gene conversion [9, 10], often carry essential genes [1, 2], and can be conserved for millions of years leading to sharing of similar structures between species [4, 11]. However, there is also turnover of palindromes between species and frequent structural rearrangement within species [4, 11, 12]. Despite their importance for health as carriers of essential proteins such as histones [8], for fertility [13], and in facilitating somatic rearrangements during the development of cancers [14], the processes underlying palindrome loss and gain in humans remain unclear.

We have investigated structural rearrangements in one palindrome, P8 carrying the *VCY* genes [15], located on the human Y chromosome. The male-specific region of the Y chromosome does not recombine and shows the lowest level of population variation of any human chromosome [16]. Yet it carries extensive overall structural variation (reviewed in [12, 17]), mostly mediated by NAHR but with a minority of events involving non-homologous rearrangements [18]. In addition, gene conversion, as well as maintaining sequence identity between palindrome arms as mentioned above, can lead to exchange of information with the X chromosome [10] and can itself create structural variation [19].

In the reference sequence, the small P8 palindrome has arm lengths of 38.0 and 37.4 kb, each with one *VCY* copy, together with a central spacer of 3.4 kb (78.8 kb in total) [1], and it is one of the palindromes that has a similar structure size and in chimpanzee [3, 4]. Nevertheless, it was shown to vary in copy number in humans among the 1000 Genomes Project phase 3 males [20, 21], where it was initially referred to as “CNV Region 4.” We demonstrate here using a range of techniques that complex structural variation in this palindrome can be identified that extends beyond changes to the number of copies of the reference sequence amplicon, and characterize this variation at the sequence level. The results reveal drastic structural changes ranging from loss of half of the palindrome regenerating a unique sequence to the formation of an entire new palindrome nearby.

## Results

### The *VCY*-containing palindrome P8 is structurally variable in the human population

Copy number variation in P8 was previously surveyed in 1234 worldwide samples by searching for an increase or decrease in read depth compared to the average normalized Y-chromosomal read depth for each sample, validated using array-CGH intensity, and also in one of the samples with increased read depth using alkaline lysis fiber-FISH [20]. There were in all 12 samples with non-reference copy numbers, and these were re-confirmed by manual inspection of the read depth and array-CGH data and placed on the Y-SNP-based phylogeny, where they fell on eight distinct branches (see Table 1).

**Table 1:** Characteristics of structural rearrangements involving palindrome P8

Cell line	VCY CN	Hg	Rearrangement summary	Novel junction structure	Data types	Similar samples	Possible mechanisms
HG00742	1	R1b-L11	37.8-kb deletion removing one P8 arm	14-bp insertion	Fiber-FISH 10x		NHEJ
HG01781	3	J2a-M410	31.5-kb inverted duplication expanding P8 to 113.8 kb	Recombination within 92-bp duplicated region	Fiber-FISH 10x	HG01991	NAHR or BIR
HG02390	3	O2-K18	191.3-kb tandem duplication distal to P8	Recombination within 2-bp microhomology region	Fiber-FISH 10x	HG01031, HG02401, HG00982	MMBIR
HG01377	4	E1b-M35	Complex formation of additional 436.7-kb new palindrome	Recombination within 5-bp microhomology region	Fiber-FISH 10x		MMBIR
HG01097	3	G-M201	158.1-kb tandem duplication distal to P8	Recombination within 6-bp microhomology region	Fiber-FISH 10x		MMBIR
HG04131	3	R1a-Z93	70.1-kb tandem duplication distal to P8	Complex recombination within 5.1-kb duplicated region	Fiber-FISH 10x		NAHR or BIR
NA18953	3	O2b-M176	60.5-kb tandem duplication proximal to P8	Recombination within 5.1-kb duplicated region	Fiber-FISH 10x		NAHR or BIR
HG00707*	3	O3-M122	60.5-kb tandem duplication proximal to P8	–	Fiber-FISH		–

*Abbreviations*: *CN* copy number, *Hg* haplogroup (haplogroup defined from previous work [16]), *NHEJ* non-homologous end joining, *NAHR* non-allelic homologous recombination, *BIR* break-induced replication, *MMBIR* microhomology-mediated break-induced replication

*HG00707 showed a similar fiber-FISH structure to NA18953 but was not sequenced using 10x Genomics Linked-Read sequencing

**Fig. 1:**
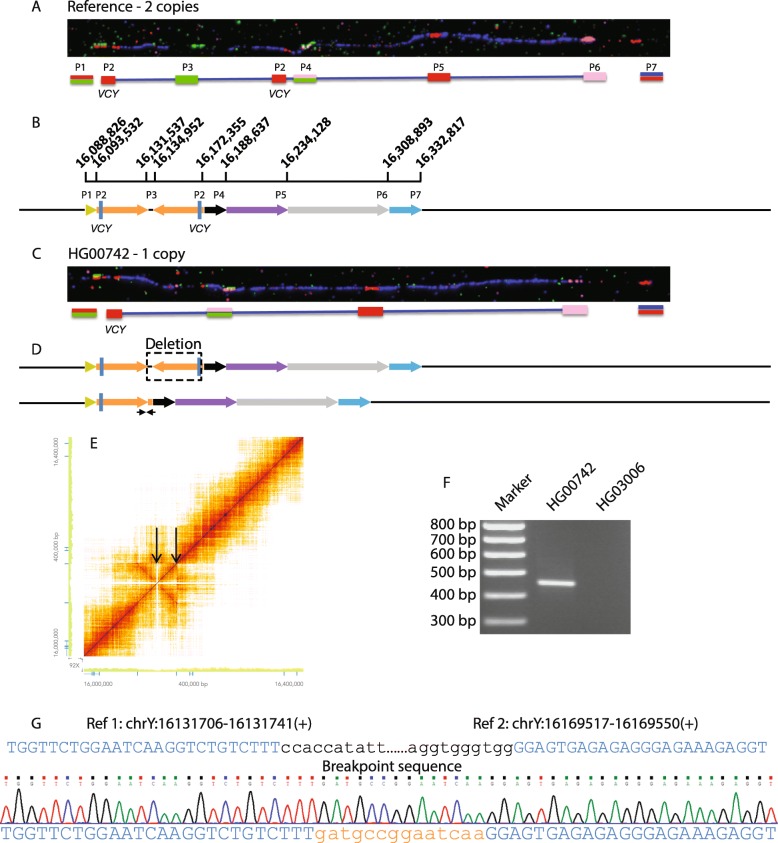
Palindrome death. **a** Schematic representation of the reference sequence for P8 and its surrounding regions. The inverted orange arrows show P8, with the *VCY* genes indicated by blue boxes. The remaining colored arrows represent sections of the flanking sequences that facilitate interpretations of the rearranged structures shown in other figures. **b** Representative fiber-FISH image of HG000096, showing the reference sequence for palindrome P8 and its surrounding regions. Seven PCR probes (1–7) together with the BAC clone RP11-53 K10 (blue) produce the fiber-FISH signals shown schematically above the fiber-FISH image. **c** Corresponding fiber-FISH image for HG00742 carrying a deletion of one P8 arm and the central spacer and lacking one red and green signal. **d** Representation of the location of the deleted section on the reference sequence (top) and resulting deleted structure (bottom). The small arrows show the location of the breakpoint PCR fragment. **e** 10x Linked-Read sequencing results represented as a Loupe file. The green axes show the read depth, and the intensity of the heat-map the extent of barcode sharing. Read depth is reduced across P8 due to deletion of the region between the black arrows and dispersal of the remaining reads across both palindrome arms. **f** Breakpoint PCR showing amplification in the deleted sample HG00742, but not in HG03006 with the reference structure. **g** Sanger sequence of the breakpoint showing (top) two regions of the reference sequence with nucleotides present in the breakpoint sequence shown in upper case blue and nucleotides absent in lower case black; (center) the sequence trace; and (bottom) nucleotides matching the reference sequence in upper case blue and novel nucleotides in lower case orange

For the present study, we chose one cell line with a non-reference copy number from each of the eight phylogenetic branches for further investigation of the P8 structure. This entailed multicolor fiber-FISH using seven PCR products as probes to define the gross new structures, sequencing using 10x Genomics Linked-Read libraries for seven of them, followed by read mapping or de novo assembly to identify junctions at the sequence level, and breakpoint PCR to validate the novel junctions (see the “Methods” section and Fig. 1). As a result, we achieved detailed understanding of the rearranged structures, which in turn suggested a diversity of mechanisms for their generation. We next present four examples of different types of structural variant; a summary and further details of these and the other four samples are reported in Table 1 and Additional file 1: Figure S1, Figure S2, Figure S3, Figure S4 and Additional file 2.

### Palindrome death by deletion of one palindrome arm

We visualized the structure of P8 and its surrounding regions via fiber-FISH using a BAC clone to mark the general region, plus a set of seven probes chosen to cover key landmarks, labeled with different combinations of three haptens (biotin, dinitrophenol, digoxingenin) and one fluorophore (Cy5). In sample HG00096 (all samples used in this study are referred to by the name beginning with “HG” or “NA” followed by five numbers, used by the 1000 Genomes Project) which matches the reference sequence P8 copy number, we see eight signals in the locations predicted by the reference sequence (Fig. 1a, b). The seven probes produce eight signals because one of them (probe 2, the short red signal labeled *VCY* in Fig. 1a marks the *VCY* gene, which is present in two copies, one close to each end of P8.

In HG00742, with a decreased read depth in P8 compared to its Y-chromosomal average, one of the red *VCY* signals and the green signal marking the 3.4-kb spacer between the two P8 arms are missing, but the other signals retain their relative positions (Fig. 1c). This suggested a deletion of the spacer plus most or all of one of the two P8 arms including *VCY*, without alteration of the surrounding sequences (Fig. 1d). This conclusion was confirmed by examining 10x Genomics Linked-Read sequence data mapped to the reference sequence, showing that the P8 region has decreased read depth compared to the average and appears as a single-copy sequence (Fig. 1e). Examination of a de novo assembly of the HG00742 genome in the region of the deletion predicted by the fiber-FISH revealed that sequences 37.8 kb apart in the reference sequence lay close together, consistent with the deletion of the 3.4-kb spacer plus most of the 37.4-kb P8 arm. PCR primers flanking the deletion produced a product in HG000742 carrying the deletion, but not in HG03006 with the reference structure (Fig. 1f), and sequencing of the PCR product revealed a structure consistent with a simple deletion together with an insertion of 14 bp of novel sequence at the breakpoint (Fig. 1g).

Thus, in HG00742, deletion has removed most of one P8 arm, generating a structure that is no longer palindromic—death of the palindrome.

### Palindrome expansion by duplication of flanking sequences

**Fig. 2:**
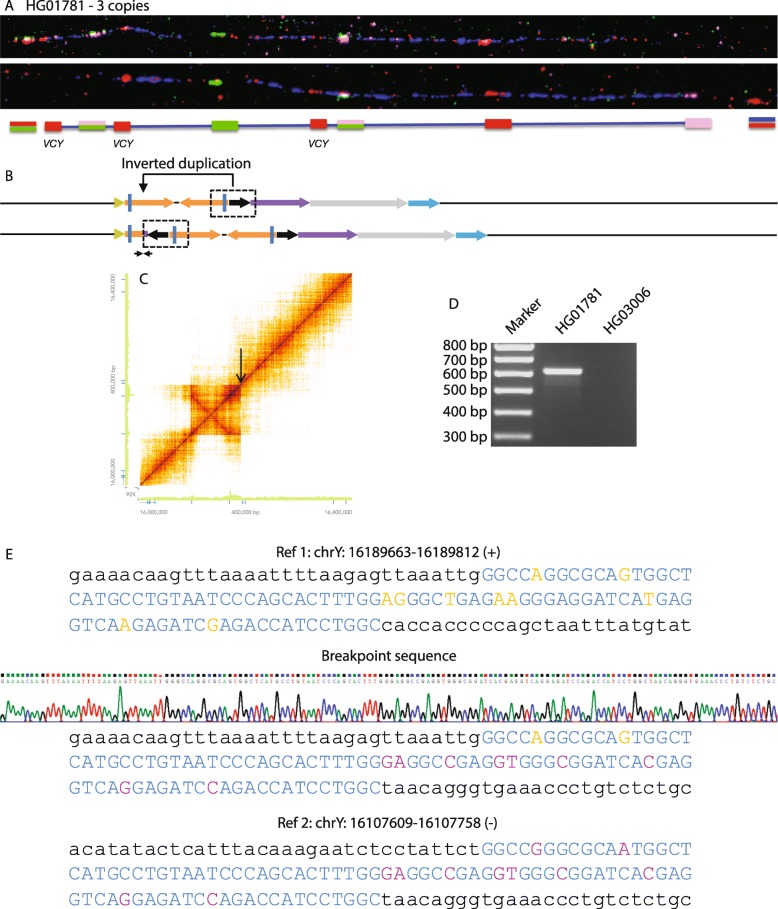
Palindrome expansion. **a**–**e** of Figs. 2, 3, and 4 show results from samples with different structural variants that correspond to sections **c**–**g** of Fig. 1. **a**, **b** Fiber-FISH image for HG01781 showing an additional copy of the short red and green+pink signals, and genomic interpretation. **c** Loupe file showing increased read depth in distal P8 and the distal flanking sequence, with a junction at the location of the black arrow. **d** Breakpoint PCR amplifies a fragment in HG01781, but not in HG03006. **e** The top and bottom sections show two regions of the reference sequence with a 92-bp repeated sequence indicated in upper case blue, except for differences between the two copies which are in orange and purple, respectively. The middle section shows the breakpoint sequence trace revealing recombination between the two 92-bp repeats

HG01781 was one of six samples examined with increased P8 read depth, compared to their Y-chromosomal averages, that indicated three copies of *VCY*. Fiber-FISH demonstrated the presence of a third short red signal, confirming that there were three *VCY* genes, and also revealed duplication of the green+pink signal which is located immediately distal to P8 (and close to *VCY*) in the reference sequence (Fig. 2a). These signals were duplicated at the proximal end of P8 in HG01781, suggesting an inverted duplication of distal P8 and the immediately flanking sequence, inserted into proximal P8 (Fig. 2b). 10x Genomics Linked-Read sequencing confirmed increased read depth of the sequences surrounding the distal end of P8 compared to the average in this part of the genome and identified a novel breakpoint (Fig. 2b, c, arrows), which was confirmed by subsequent breakpoint PCR and sequencing (Fig. 2d, e). The sequencing results showed that 14 kb of P8, together with the adjacent 17.5 kb of flanking sequence distal to P8, has been duplicated, resulting in three copies of the 14-kb P8 section containing *VCY* plus an expanded palindrome. One breakpoint did not generate a novel sequence; the other lay within a 92-bp duplicated region with 10 differences between the two copies, where the recombination event could be located to a 30-bp stretch of sequence identity (Fig. 2e).

The consequence of this event is to extend the total length of the P8 palindrome from 78.8 to 113.8 kb—an addition of 44% to the palindrome. HG01991 shares the same structure, as assessed by both read depth changes and amplification of the same diagnostic breakpoint PCR fragment (Table 1).

### Tandem duplication involving palindrome sequences

**Fig. 3:**
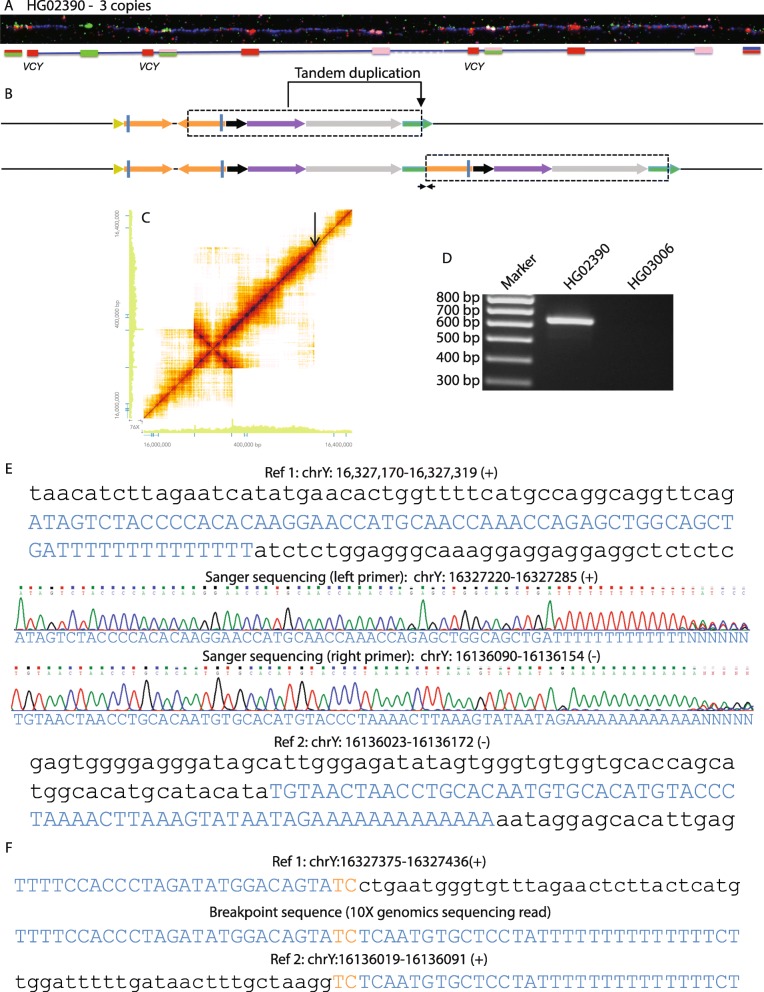
Tandem duplication of part of a palindrome. **a**, **b** Fiber-FISH image for HG02390 showing an additional copy of the short red, green+pink, and long red signals, and genomic structure. **c** Loupe file showing increased read depth in distal P8 and a long section of distal flanking sequence, with a junction at the location of the black arrow. **d** Breakpoint PCR amplifies a fragment in HG02390, but not in HG03006. **e** The top and bottom sections show two regions of the reference sequence with sections detected in the breakpoint PCR sequence in upper case blue. The middle section shows Sanger sequence traces from the breakpoint PCR product, each ending before the breakpoint in a T- or A-stretch. **f** The middle section shows a 10x Genomics sequencing read spanning the breakpoint and linking the two breakpoint Sanger sequence traces in **e**, revealing a recombination between the sequences in the upper and lower sections at the orange TC microhomology breakpoint

The other four samples with three copies of *VCY* could be explained by tandem duplications, although the duplicated region was distinct in at least three cases. Here, we present one example of them, HG02390; the others are described in Additional file 1: Figure S1, Figure S2, Figure S3. In HG02390, one short red *VCY* probe plus the three probes immediately distal to P8 was duplicated and inserted between the pink probe and red+blue probe (Fig. 3a). This structure suggested a simple tandem duplication of ~ 190 kb extending from within the distal P8 arm into the flanking sequence, an interpretation confirmed by 10x Genomics Linked-Read sequencing which revealed increased read depth over this length and a novel breakpoint-spanning sequence read (Fig. 3a, c, d). The sequence of this breakpoint (Fig. 3e) revealed recombination at 2 bp of microhomology, TC (Fig. 3f), and refined the length of the tandem duplication to 191.3 kb (Table 1).

Three additional samples, HG01031, HG02401, and HG00982, share the same structure according to their read depth estimates and amplification of the same diagnostic breakpoint PCR fragment. Two of them, HG01031 and HG00982, were also confirmed to have the same structure by fiber-FISH (Additional file 1: Figure S4).

### Palindrome birth

**Fig. 4:**
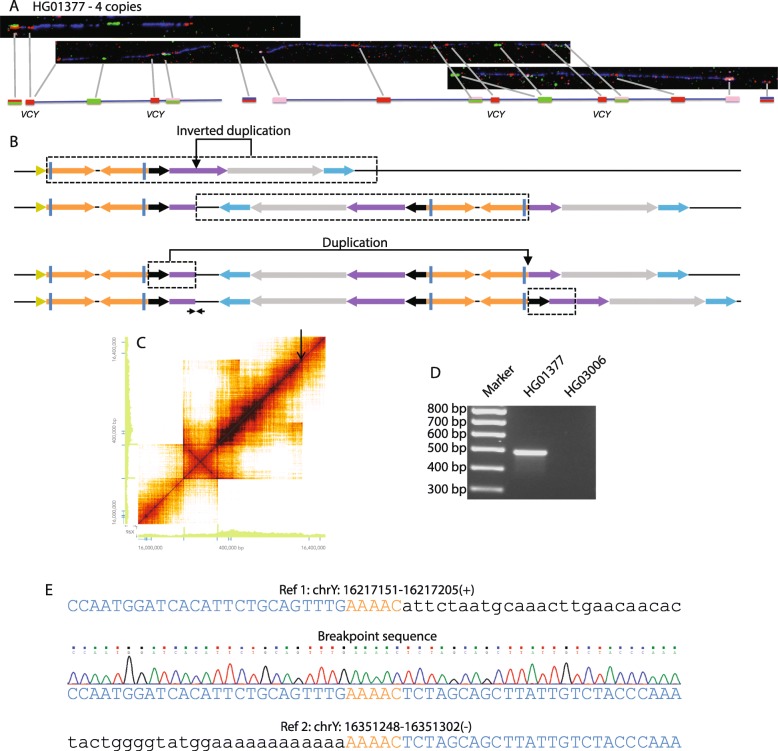
Palindrome birth. **a** Fiber-FISH image for HG01377 showing a group of multiple new signals distal to P8. Because of the length spanned by these signals, a composite of three fibers is shown, with the correspondences indicated by lines. **b** Genomic interpretation, showing that the resulting structure can be accounted for by two duplications, which could be parts of a single mutational event. **c** Loupe file showing increased read depth in distal P8 and a long section of distal flanking sequence, with a junction at the location of the black arrow. **d** Breakpoint PCR amplifies a fragment in HG01377, but not in HG03006. **e** The top and bottom sections show two regions of the reference sequence with sections detected in the breakpoint PCR sequence in upper case blue, or upper case orange for a 5-bp microhomology region at the breakpoint. The middle section shows the Sanger sequence trace from the breakpoint PCR product

HG01377 showed an increased read depth of P8 compared to its Y-chromosomal average that suggested the presence of four copies of *VCY*. Fiber-FISH confirmed that there were four short red *VCY* gene signals, two of which lay in their original context, with the other two also in a context similar to the original, but 224 kb away and, judging from the flanking sequences, being inverted (Fig. 4a). No simple rearrangement event could explain the new structure, but a more complex series of events resulting in duplication of the entire P8 palindrome plus a large region of distal flanking sequence could do so (Fig. 4b). 10x Genomics Linked-Read sequencing showed increased read depth of P8 and almost 180 kb of distal sequence compared to the average in the surrounding regions and allowed a novel junction to be identified (Fig. 4b, c, arrows), which was confirmed by breakpoint PCR and sequencing (Fig. 4d, e). The junction sequence could be explained by recombination at a 5-bp stretch of microhomology AAAAC (Fig. 4e) and showed that ~ 178.9 kb of distal flanking sequence had been incorporated into each side of the new structure, resulting in an additional palindrome with a total length of 436.7 kb (Fig. 4b).

The complex set of events in HG01377 thus result in the retention of the original P8 structure and the birth of an entire new palindrome more than twice the size of the original P8.

### Structures of palindrome rearrangement breakpoints

We have been able to define the structures of seven rearrangements at both the gross and breakpoint sequence levels (Figs. 1, 2, 3, and 4; Additional file 1: Figure S1, Figure S2, Figure S3; and Additional file 2: Supplementary notes). The gross structures consist of one deletion (HG00742, Fig. 1), one inverted duplication (HG01781, Fig. 2), four tandem duplications (HG02390, HG01031, HG02401, and HG00982, Fig. 3 and Additional file 2: Figure S1, Figure S2, Figure S3), and one complex event (HG01377, Fig. 4); these are summarized in Table 1. The duplications all have the characteristic that one end creates a novel sequence junction while the other end does not.

The junction of the deletion in HG00742 revealed simple loss of sequence together with insertion of 14 bp of novel sequence at the breakpoint (Fig. 1g). The other junctions fell into two categories. Three junctions (HG02390, HG01377, and HG01097) showed 2–6 bp of microhomology at the breakpoint, while the other three (HG01781, HG04131, and NA18953) lay in repeated sequences which have prevented the breakpoint from being identified to the bp level (Table 1). In one of the latter three, there was a complex mosaic of the two repeated sequences, identifiable by occasional sequence differences between them, rather than a clean break (HG04131, Additional file 1: Figure S2).

These structures provide information about the likely mechanisms of their formation, which we consider in the “Discussion” section.

### *VCY* copy number variant selection or neutrality inferred from their locations on the Y-chromosomal SNP phylogeny

**Fig. 5:**
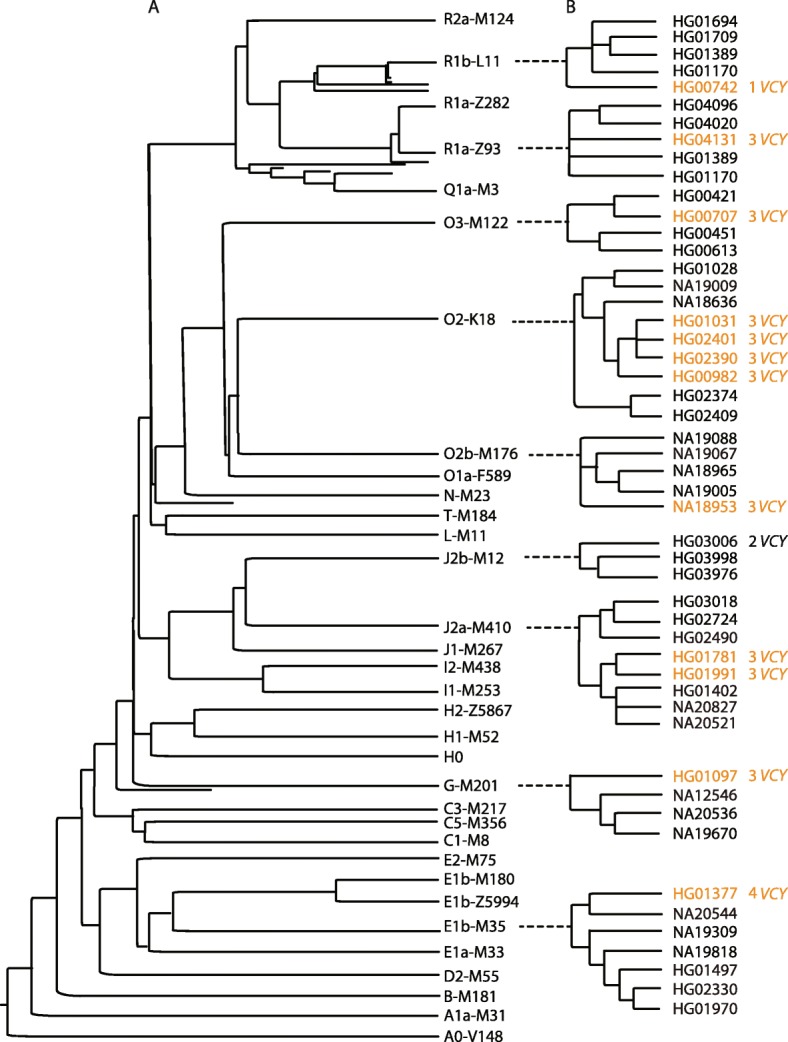
Location of *VCY* copy number variants on the Y-chromosomal phylogeny. A Y-chromosomal phylogeny at the level of the major haplogroups. The branching order is shown, but the line lengths have no meaning. B Clusters of lineages including each of the *VCY* CNVs identified (brown) and the sample representing the reference sequence (HG03006). These clusters lie within the major haplogroup indicated by the dotted line

The 12 samples with *VCY* copy numbers other than two were carried by eight different Y haplogroups dispersed throughout the SNP-based phylogeny constructed for the same set of Y chromosomes (Fig. 5). Six samples each correspond to a single haplogroup, while the remaining six fell into two haplogroups: two in Y haplogroup J2a-M410 and four in O2-K18. The breakpoint PCRs described in the previous sections showed that all samples within each of these two groups shared the same breakpoint, and the branch lengths in the tree indicated that each of these two haplogroups had a recent common ancestor, ~ 12,400 or 1500 years ago, respectively. These observations provide strong evidence that there were thus eight distinct mutational events, at least two of which were able to expand in the population, demonstrating that their carriers are fertile and thus not strongly selected against.

We further explored the possibility that weak negative selection, or indeed positive selection, might be acting on *VCY* copy number variants by comparing their population frequency distribution with those of synonymous variants (assumed to be neutral) and non-synonymous variants (assumed to be on average subject to weak negative selection) previously identified in the same dataset [20]. Variants of each type (synonymous, non-synonymous, *VCY*) were classified into three frequency groups: *n* = 1 (singletons), *n* = 2 (doubletons), and *n* > 2, and compared using a chi-squared test with two degrees of freedom. Synonymous and non-synonymous variant frequencies analyzed in this way were significantly different (*p* = 0.00058), as expected. *VCY* variant frequencies were not significantly different from synonymous variant frequencies (*p* = 0.606), but were significantly different from non-synonymous variant frequencies (*p* = 0.025). *VCY* structural variants are therefore less strongly affected by negative selection than non-synonymous variants are and, within the limitations of this test, are consistent with neutrality.

## Discussion

Our survey of P8 structural variation in the general population has revealed substantial variation at this locus and has provided some insights into *VCY* gene function, rearrangement mechanisms, and longer-term palindrome evolution. Since our work was based on cell lines, we first consider whether the structural variants we describe are likely to have arisen in the cell lines as a result of somatic mutations, or whether they were more likely to have been present in the sample donors. Those shared by two or more samples forming a consistent clade in the Y-chromosomal phylogeny have been inherited and thus must have been present in the sample donors. The six variants present in single samples are more difficult to assess, but we see no evidence for mosaicism in any of them, and it would be more surprising for all of them to have become fixed in the cell cultures than for them to have been pre-existing in the sample donors. We therefore conclude that they are most likely germ-line variants.

The *VCY* genes are members of a gene family that also contains X-chromosomal members designated *VCX*, all with expression reported exclusively in male germ cells [15]. VCY proteins have been detected largely in germ cell nuclei, and expression in cultured COS7 cells suggested localization in nucleoli, where they may interact with the ribosomal protein PO [22]. These biochemical properties, however, do not explain the biological function of a Y-encoded testis-specific family member in the whole organism. No deletions that remove only *VCY* genes have been reported, so genetic analysis has not helped to define VCY function. Studies of *VCY* evolution have suggested an origin after the divergence from macaque ~ 25 million years ago [23] and demonstrated an extraordinarily high 4.3% nucleotide sequence divergence between the human and chimpanzee coding regions, compared with a more typical 1.6% in the introns [4]. This divergence leads to a dN/dS ratio of 3.1 [3], and although ratios > 1 are often taken to indicate positive selection for rapid amino acid change, the high *VCY* dN/dS ratio results mainly from structural alterations towards the 3′ end of the coding region that might instead indicate non-essentiality in chimpanzees [3]. The maintenance of an open reading frame [1, 15] and expression [15, 22] in humans, however, suggest functional importance in our species. The current study is consistent with this conclusion, since, despite extensive variation in copy number, demonstrating the mutability of the region and tolerance of different copy numbers between one and four plus the four apparently functionally similar *VCX* genes, at least one *VCY* copy is always retained.

Mechanisms for generating structural variation have been divided into recurrent and non-recurrent classes, with recurrent mutations often associated with repeated sequences [24]. The rich repeated-sequence environment of the Y chromosome means that recurrent rearrangements are particularly common and account for most well-studied copy number variants found in Y chromosomes, but non-recurrent rearrangements are also known [18]. The eight groups of variants investigated here, despite being ascertained for P8 copy number variation alone, are (with the possible exception of NA18953 and HG00707) all different from one another and thus non-recurrent in our sample. The male-specific region of the Y chromosome, in which P8 is located, does not recombine, and so mutational mechanisms are further limited to those that do not require recombination.

Breakpoint structures provide the main information (albeit indirect) about the likely underlying mutational mechanisms. Key factors are the presence or absence of homology at the breakpoint, and if homology is present, its length. Non-homologous end joining (NHEJ) usually generates simple, blunt junctions between regions without homology; however, the insertion of random nucleotides can also be observed at breakpoints [24]. NHEJ is thus the most likely mutational mechanism for the deletion in HG00742. A second group of three structural variants are associated with low-copy repeats between 92 bp and 5.1 kb in length (HG01781, HG04131, and NA18953). The recombination events occur in segments of sequence identity within divergent copies of the repeat, and in one case (HG04131), the sequence differences between the two 5.1-kb repeats form a mosaic pattern. These three structural variants are likely to have arisen by non-allelic homologous recombination (NAHR) or break-induced replication (BIR) [24]. The third group of structural variants, also with three members (HG01097, HG02390, and HG01377), are characterized by 2–6 bp of microhomology at the breakpoint, implying a mutational mechanism involving microhomology-mediated break-induced replication (MMBIR) [24]. For HG01377, the complex rearrangement could be explained by two template switches during one MMBIR event.

## Conclusions

We have characterized the P8 structural variation in healthy participants in the 1000 Genomes Project, showing that it results in diverse structures carrying from one to four copies of *VCY*. In some cases, phylogenetic clusters of the same rearrangement were observed, indicating successful transmission in the population over several generations and thus fertility of the carriers, while the frequency distribution of the variants in the population is distinguishable from that of non-synonymous variants, but not from that of synonymous variants. These P8 structural variants therefore do not have strongly deleterious consequences and are consistent with evolutionary neutrality and provide examples of the pool of variation that could potentially predominate in future Y chromosomes. Strikingly diverse structures are present, showing that P8, and probably other palindromes, can readily lose their palindromic structure, or change it drastically, most likely in a single mutational event. The most dramatic of these creates a new palindrome with arm length over 200 kb, making it the fourth largest on the Y chromosome in contrast to P8, which is the smallest except for P7 [1]. Such events suggest that detailed investigations of structural variation in other palindromes are warranted, and readily explain how palindrome structures change radically over evolutionary timescales.

## Methods

### Multiplex fiber-FISH

Thirteen samples were examined: HG00096 with *VCY* copy number of 2, representing the reference sequence structure, and HG00742, HG01781, HG01991, HG01097, HG02390, HG01031, HG02401, HG00982, HG04131, NA18953, HG00707, and HG01377 with copy numbers that are different from the reference genome. Lymphoblastoid cell lines were purchased from the Coriell Institute for Medical Research (https://www.coriell.org/). PCR probes were amplified by long-range PCR using the primers listed in Additional file 3: Table S1, with the annealing temperatures suggested by the manufacturer. The PCR products were run on an agarose gel to confirm the length of the amplified fragment and purified by gel extraction using a QIAquick Gel Extraction Kit according to the protocol indicated by the manufacturer. Six out of seven probes were produced as individual PCR products, using a Forward and Reverse primer (Additional file 3: Table S1); the seventh was produced as a mixture of two separate PCR amplifications, both of which were obtained using a single PCR primer annealing on both arms of the P8 palindrome, thus spanning the palindrome spacer. The BAC clone RP11-53 K10 was used to highlight the target region. The preparation of probes and single molecular fibers as well as multiplex fiber-FISH were carried out as described previously [25].

### 10x Genomics Linked-Read sequencing and data processing

Eight of the samples used for fiber-FISH validation (omitting HG00707, which had a similar fiber-FISH pattern to NA18953) were processed using 10x Genomics Chromium technology V1 [26], producing linked reads from long single molecules which can be used for both phasing and de novo assembly. The molecular length of the DNA was measured using the Agilent TapeStation and confirmed to be greater than 50 kb and thus suitable for Chromium library construction. The libraries were sequenced on single Illumina HiSeqX lanes. The sequencing reads were processed with the 10x Genomics Long Ranger software (version 2.1.2) to generate a phased BAM file for each sample. They were also used to generate de novo assemblies using 10x Supernova software version 1.1.2 with the default settings. The contigs from the de novo assembly were treated as single-end reads and mapped to reference sequence 1000Genomes_hs37d5 using BWA MEM version 0.7.17-r1188 to produce contig BAMs. Both phased BAMs from Long Ranger and contig BAMs were imported into the Integrative Genomics Viewer (IGV) to manually view details of the mapped reads for identifying breakpoints.

### Breakpoint identification from 10x Genomics sequence data

The contigs from the Supernova assemblies allowed the breakpoint of the deletion in HG00742 to be identified. For the other samples, where the breakpoints lay in repeated regions, the Long Ranger analysis output was used and the details are shown in Additional file 2. In summary, the Loupe file from each sample was imported into the Loupe genome browser from the Matrix view, and structural variation visualization in the region of 16.0–16.4 Mb was used to identify the specific location of the breakpoints by comparing the pattern with the reference sequence. Then, the BAM files from the same sample were imported into the IGV browser to identify the location of any sudden change in read depth or unusual reads, such as split reads, large insert size, or reads clipped at the same position. This approach identified breakpoints in five samples. In NA18953, no informative reads were found and the breakpoint could only be narrowed down to a ~ 5-kb region in this way.

### Experimental validation

For all samples except NA18953 and HG04131, primers could be designed to amplify the breakpoint identified from the 10x Genomic data (Additional file 3: Table S2). After amplification and testing for specificity of the product to the relevant sample by gel electrophoresis, the PCR products were Sanger sequenced by Eurofins UK.

For NA18953, we first designed four pairs of primers flanking the 5.1-kb repeated sequence (Table 1) to specifically amplify the relevant copy of the repeat. One pair produced a 6.0–7.0-kb fragment (Additional file 3, Table S3). Then, to identify the breakpoint, we designed another 20 sequencing primers to initiate Sanger sequencing through the whole 5.1-kb region. The exact breakpoint was successfully identified by one of these sequencing primers (Additional file 3: Table S3).

For HG04131, we designed primers inside the 5.1-kb repeated sequence (Table 1) and with one combination saw specific amplification (of a ~ 3.5-kb product) as well as two unspecific bands also observed in HG03006 (Additional file 1: Figure S2). We gel-purified the specific band and designed six Sanger sequencing primers spanning it (Additional file 2: Table S4). The breakpoint was identified by one of these primers.

The Sanger sequences were viewed and analyzed using Chromas (version 2.6.6, http://technelysium.com.au/wp/).

## Supplementary information

**Additional file 1:** **Figure S1.** Tandem duplication in HG01097. **Figure S2.** Tandem duplication in HG04131. **Figure S3.** Tandem duplication in NA18953. **Figure S4.** Fiber-FISH results for HG00707, HG01031 and HG00982. (PDF 689 kb)

**Additional file 2:** Supplementary notes for breakpoint identification. (PDF 4747 kb)

**Additional file 3:** **Table S1.** PCR primers used for amplification of fiber-FISH probes. **Table S2.** PCR primers for breakpoints identification. **Table S3.** Sanger sequencing primers for NA18953 breakpoint PCR product. **Table S4.** Sanger sequencing primers for HG04131 breakpoint PCR product. (XLSX 14 kb)

**Additional file 4:** Review history. (DOCX 19 kb)

## Data Availability

Sequences of breakpoint PCR products are available from the European Nucleotide Archive under accession code ERP015752 and grp 7215690 [27, 28].
