# Supplementary material for: Birth, expansion, and death of VCY-containing palindromes on the human Y chromosome
Source: Genome Biol. 2019 Oct 14;20:207. doi: 10.1186/s13059-019-1816-y (PMC6790999; doi:10.1186/s13059-019-1816-y)

Figure S1. Tandem duplication in HG01097

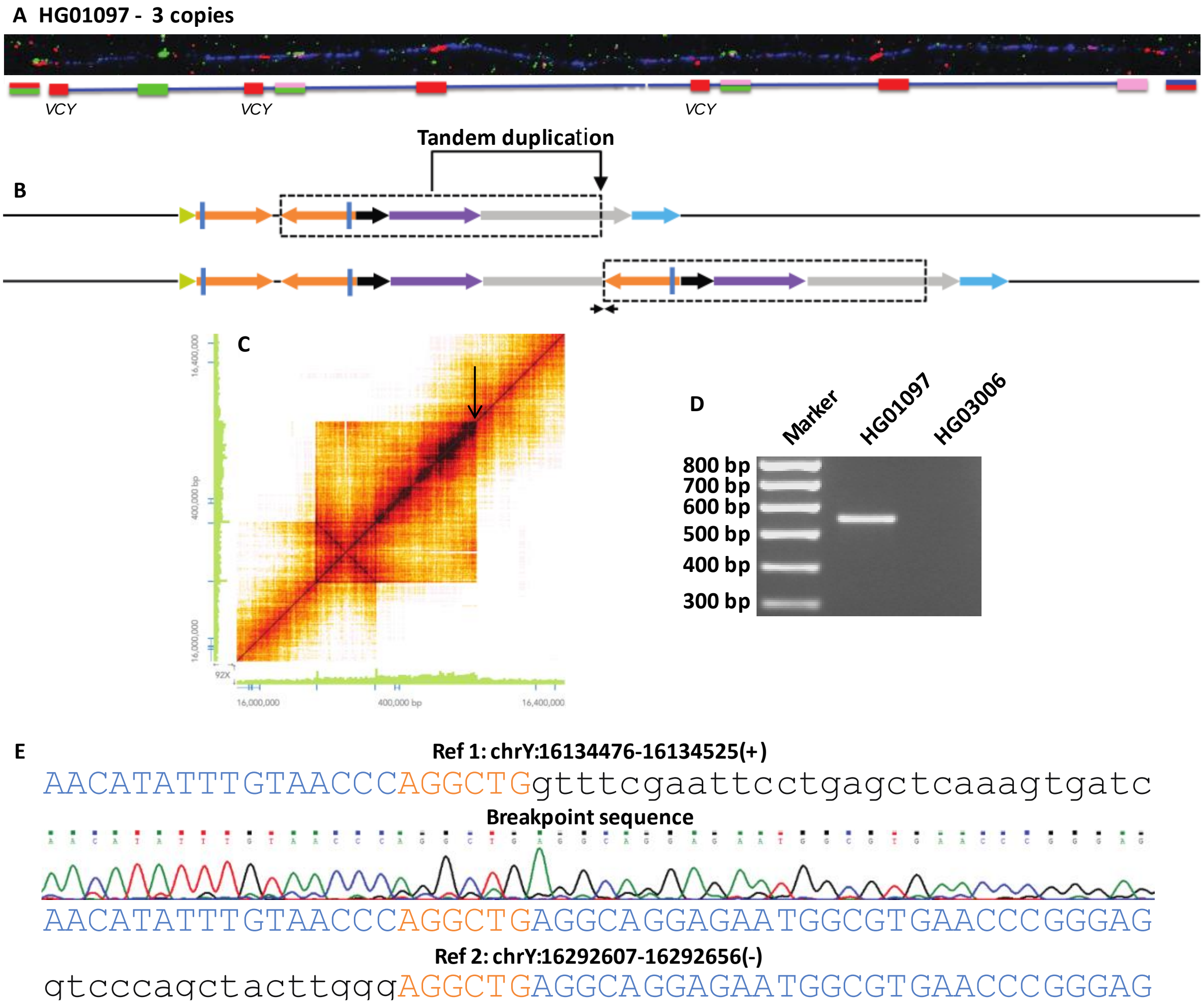

Figure S2. Tandem duplication in HG04131

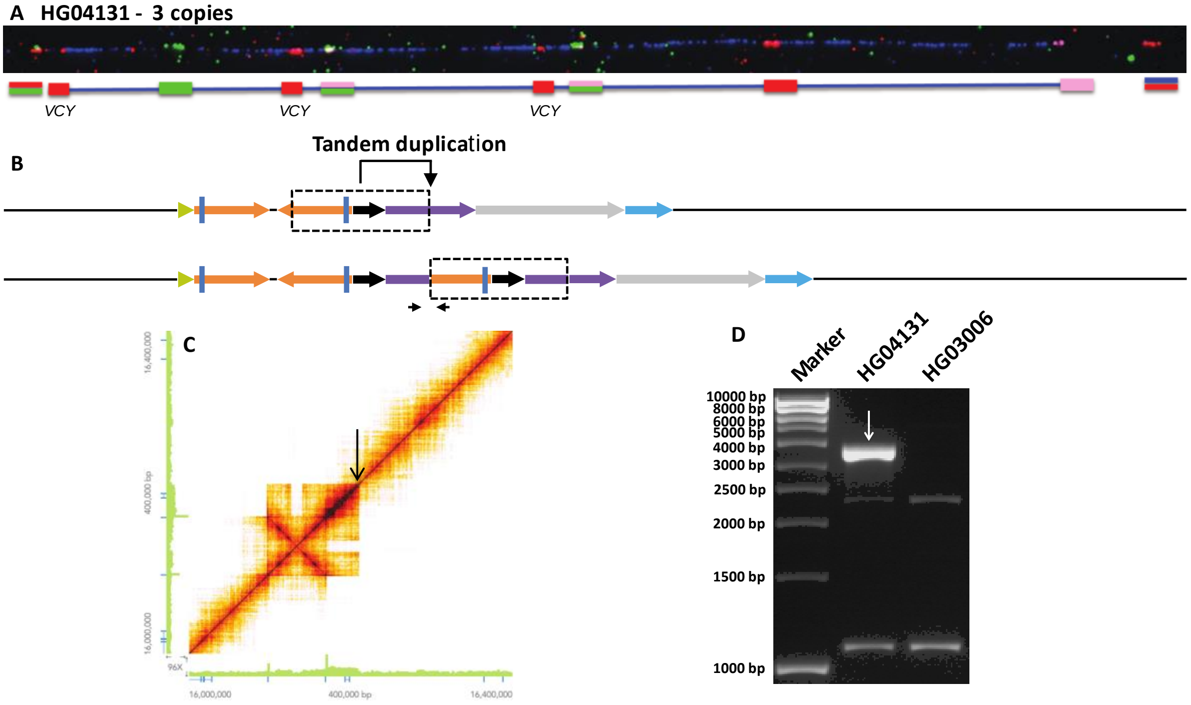

E

Ref 1: chrY: 16139889-16140438(-)

ATCAGACTAACAGTGGATCTCTCGGCAGAAACCCTACAAGCCAGAAGAGA
GTGGGGGCCAATATTCAACATTCTTAAAGAAAAGAATTTTCAACCCAGAA
TTTCATATCCAGCCAAACTAAGCTTCATAAGCGAAGGAGAAATAAAATAC
TTTGCAGACAAGCAAATGCTGAGAGATTTTGTCACCACCAGGCCTGCCCT
AAGAGAGCTCCTGAAGGAAGCGCTAAACATGGAAAGGAACAACCGCTACC
AGCCGCTGCAAAATCATGCCAAAATGTAAAGACCATCAAGACTAGGAAGA
AACTGCATCAACTAACGAGCAAAAGAACCAGCTAACATCATAATGACAGG
ATCAAATTCACACATAACAATATTAACTTTAAATGTAAATGGACTAACTG
CTCCAATTAAAAGACACAGACTGGCAAATTGGATAAAGAGTCAAGACCCA
TCAGTGTGCTGTATTCAGGAAACCCATTTCACGTGCAGAGACACACATAG
GCTCAAAATAACAGAATGGAGGAAGATCTACCAAGCAAATGGAAAAGAAA

Breakpoint sequence

ATCAGACTAACAGTGGATCTCTCGGCAGAAACCCTACAAGCCAGAAGAGA
GTGGGGGCCAATATTCAACATTCTTAAAGAAAAGAATTTTCAACCCAGAA
TTTCATATCCAGCCAAACTAAGCTTCATAAGCGAAGGAGAAATAAAATAC
TTTGCAGACAAGCAAATGCTGAGAGATTTTGTCACCACCAGGCCTGCCCT
AAAAGAGCTGCTGAAGGAAGCACTAAACATGGAAAGGAACAACCGGTACC
AGCCGCTGCAAAATCATGCCAAAATGTAAAGACCATCGAGGCTAGGAAGA
AACTGCATCAACTAACGAGCAAAATAACCAGCTAACATCATAATGACAGG
ATCAAATTCACACATAACAATATTAACTTTAAATGTAAATGGACTAAATG
CTCCAATTAAAAGACACAGACTGGAAAATTGGATAAAGAGTCAACACCCA
TCAGTGTGCTGCATTCAGGAAACCCATCTCACGTGCAGAGACACACATAG
GCTTAAAATAAAGGATGGAGGAAGATCTACCAAGCAAATGGAAAACAAA

Ref 2: chrY: 16209994-16210543(-)

ATCAGACTAACAGTGCATCTCTCGGTAGAAACTCTACAAGCCAGAAGAGA
GTGGGGGCCAATATTCAACATTCTTAAAGAAAAGAATTTTTAACCTAGAA
TTTCATATCCAGCCAAACTAAGCTTCATAAGTGAAGGAGAAATAAAATAT
TTTACAGAGAAGCAAATGCTGAGAGATTTTGTCACCACCAGGCCTGCCCT
AAAAGAGCTGCTGAAGGAAGCACTAAACATGGAAAGGAACAACCGGTACC
AGCCACTGCAAAATCATGCCAAAATGTAAAGACCATCGAGGCTAGGAAGA
AACTGCATCAACTAACAAGCAAAATAACCAGCTAACATCATAATGACAGG
ATCAAATTCACACATAACAATATTAACTTTAAATGTAAATGGACTAAATG
CTCCAATTAAAAGACACAGACTGGAAAATTGGATAAAGAGTCAACACCCA
TCAGTGTGCTGCATTCAGGAAACCCATCTCACGTGCAGAGACACACATAG
GCTTAAAATAAAGGATGGAGGAAGATCTACCAAGCAAATGGAAAACAAA

Figure S3. Tandem duplication in NA18953

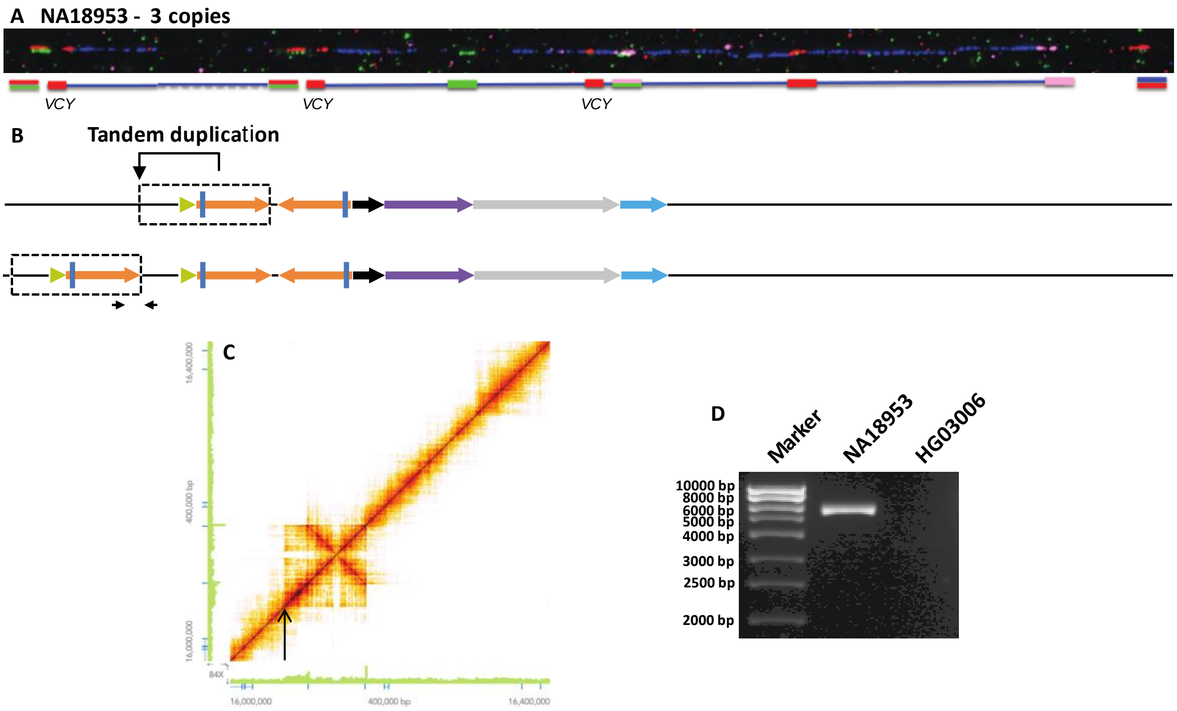

E

Ref 1: chrY :16128218-16128817(+)

AACACTTCATGCTAAAAGCTCTCAATAAATTAGGTATTGATGGGATGTAT
TTCAAAATAATAAGAGCTATCTATGACAAAGCCACAGCCAATATCATACT
GAATGGGCAAAAACTGGAAGCATTCCCTTTGAAAACTGGCACAAGACAGG
GATGCCCTCTCTCACCACTCCTATTCAACATAGGTTTGGAAGTTCTGGCC
AGGGCAATTAGGCAGGAGAAGGAAATAAAGGGTATTCAATTAGGAAAAGA
GGAAGTCAAATTGTCCCTTTTTGCAGACGACATGATTGTATATCTAGAAA
ACCCCATTGTCTCAGCCCAAAATCTCCTTAAGCTGATAAGCAACTTCAGC
AAAGTCTCAGGATACAAAATCAATGTACAAAAATCACAAGCATTCTTATA
CACCAACAACAGACAGACAGAGAGCCAAGTCATGAGGGAACTCCCATTCA
CAGTTGCTTCAAAGAGAATAAAATACCTAGGAATCCAACTTACAAGGTAT
GTGAAGGACCTCTTCAAGGAGAACTACAAACCACTGCTCAAGGAAATAAA
AGAGGACACAAACAAATGGAAGAACATTCCCTGCTCATGGGTAGGAAGAA

Breakpoint sequence

AACACTTCATGCTAAAAGCTCTCAATAAATTAGGTATTGATGGGATGTAT
TTCAAAATAATAAGAGCTATCTATGACAAAGCCACAGCCAATATCATACT
GAATGGGCAAAAACTGGAAGCATTCCCTTTGAAAACTGGCACAAGACAGG
GATGCCCTCTCTCACCACTCCTATTCAACATAGGTTTGGAAGTTCTGGCC
AGGGCAATTAGGCAGGAGAAGGAAATAAAGGGTATTCAATTAGGAAAAGA
GGAAGTCAAATTGTCCCTTTTTGCAGACGACATGATTGTATATCTAGAAA
ACCCCATTGTCTCAGCCCAAAATCTCCTTAAGCTGATAAGCAACTTCAGC
AAAGTCTCAGGATACAAAATCAATGTACAAAAATCACAAGCATTCTTATA
CACCAACAACAGACAGACAGAGAGCCAAGTCATGAGGGAACTCCCATTCA
CAGTTGCTTCAAAGAGAATAAAATACCTAGGAATCCAACTTACAAGGGAT
* *
GTGAAGGACCTCTTCAAGGAGAACTACAAACCACTGCTCAAGGAAATAAA
AGAGGATACAAACAAATGGAAGAACATTCCCTGCTCATGGGTAGGAAGAA

Ref 2: chrY: 16067637-16068236(+)

AACGCTTCATGCTAAAAACTCTCAATAAATTAGGTATTGATGGGACATAT
CTCAAAATAATAAGAGCTATCTATGACAAACCCACAGCCAATATCATACT
GAATGGGCAAAAACTGGAAGCATTCCCTTTGAAAACTGGCACAAGACAGG
GATGCCCTCTCTCACCACTCCTATTCAACATAGGTTTGGAAGTTCTGGCC
AGGGCAATTAGGCAGGAGAAGGAAATAAAGGGTATTCAATTAGGAAAAGA
GGAAGTCAAATTGTCCCTTTTTGCAGACGACATGATTGTATATCTAGAAA
ACCCCATTGTCTCAGCCCAAAATCTCCTTAAGCTGATAAGCAACTTCAGC
AAAGTCTCAGGATACAAAATCAATGTACAAAAATCACAAGCATTCTTATA
CACCAACAACAGACAGACAGAGAGCCAAGTCATGAGGGAACTCCCATTCA
CAATTGCTTCAAAGAGAATAAAATACCTAGGAATCCAACTTACAAGGGAT
GTGAAGGACCTCTTCAAGGAGAACTACAAACCACTGCTCAAGGAAATAAA
AGAGGATACAAACAAATGGAAGAACATTCCCTGCTCATGGGTAGGAAGAA

Figure S4. Fibre FISH results for HG00707, HG01031, HG2041, HG01991 and HG00982

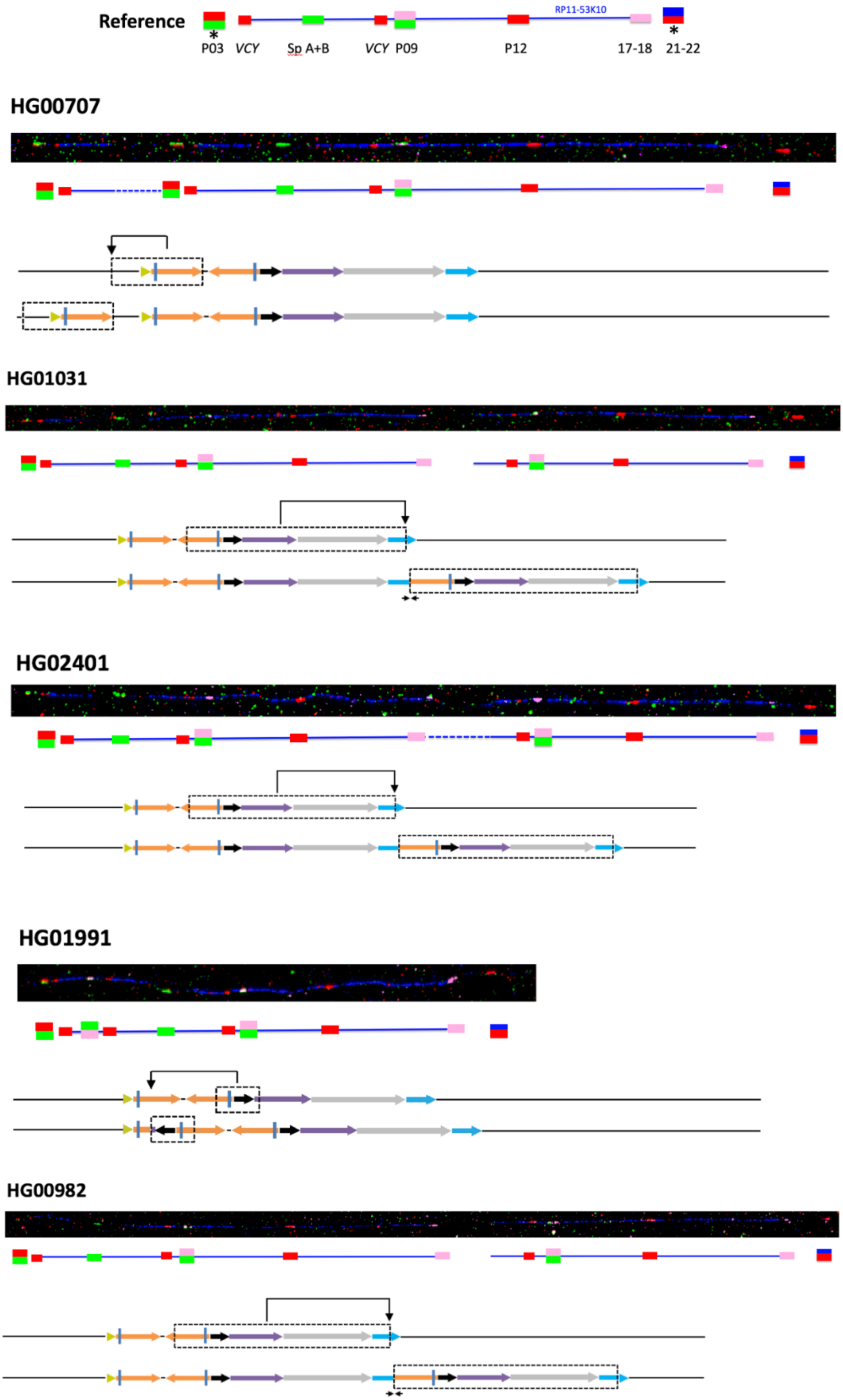

Supplement: Supplementary file 1 — Additional file 1: Figure S1. Tandem duplication in HG01097. Figure S2. Tandem duplication in HG04131. Figure S3. Tandem duplication in NA18953. Figure S4. Fiber-FISH results for HG00707, HG01031 and HG00982. (PDF 689 kb) [file 13059_2019_1816_MOESM1_ESM.pdf]
