# Supplementary material for: Birth, expansion, and death of VCY-containing palindromes on the human Y chromosome
Source: Genome Biol. 2019 Oct 14;20:207. doi: 10.1186/s13059-019-1816-y (PMC6790999; doi:10.1186/s13059-019-1816-y)
Supplement: Supplementary file 2 — Additional file 2: Supplementary notes for breakpoint identification. (PDF 4747 kb) [file 13059_2019_1816_MOESM2_ESM.pdf]

Sample with reference sequence

FibreFISH image

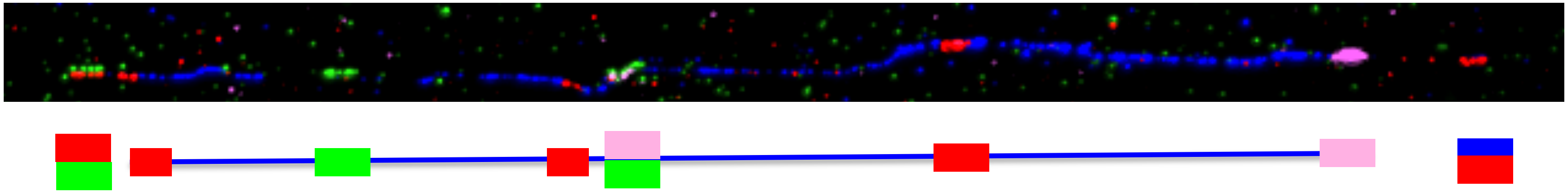

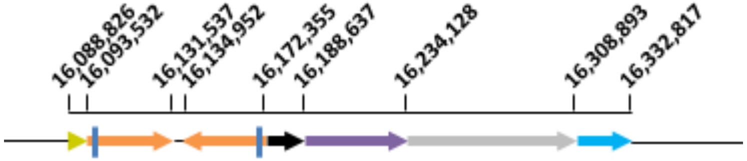

Diagram of the palindrome structure

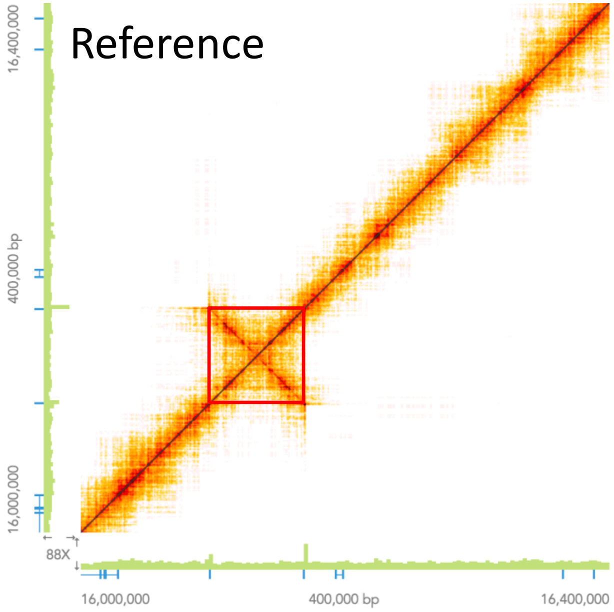

Matrix view from 10x link read loupe file.
The red box marks P8.

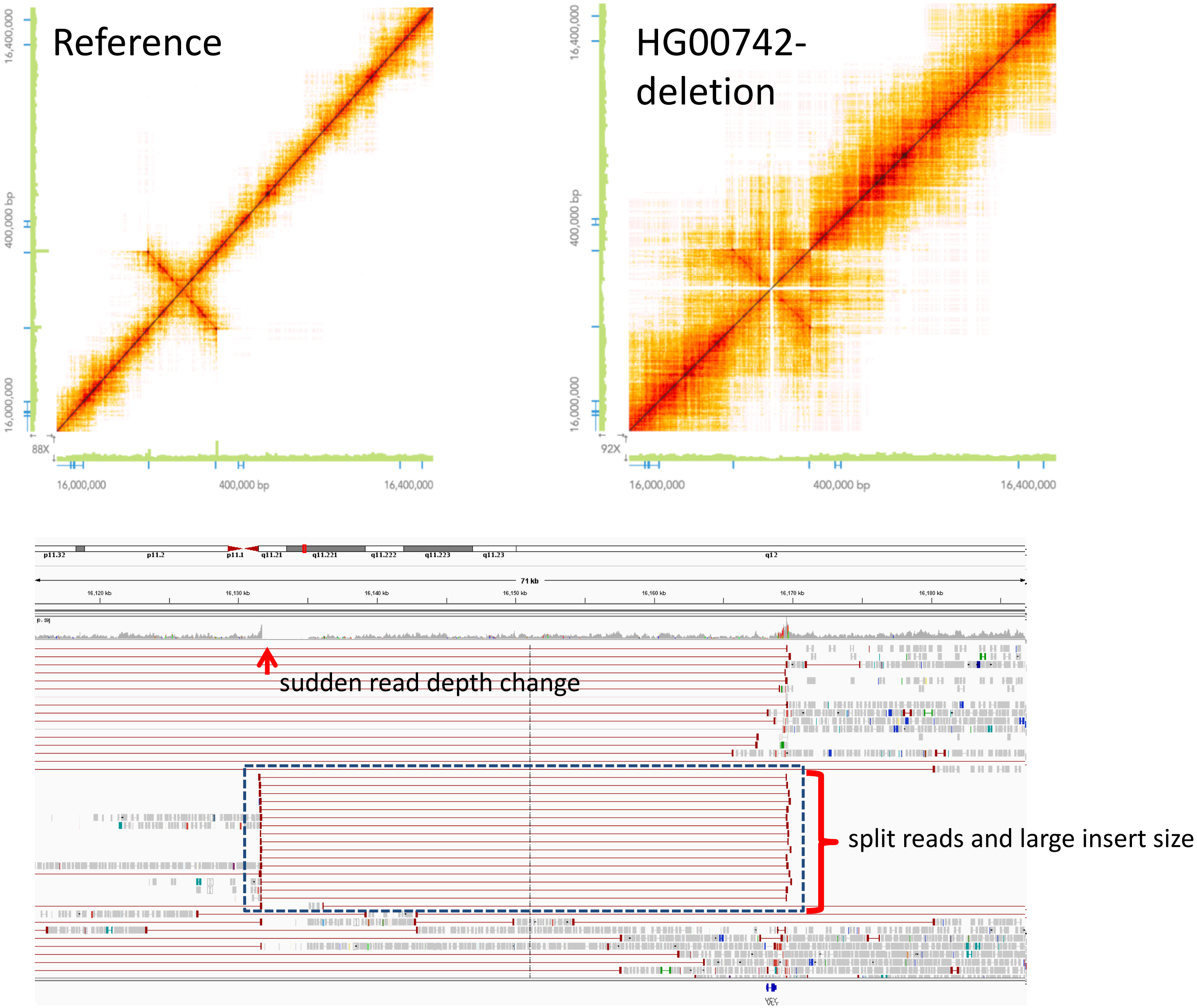
Reference
HG00742-deletion
88X
92X
16,000,000
400,000 bp
16,400,000
p11.32
p11.2
p11.1
q11.21
q11.221
q11.222
q11.223
q11.23
q12
71 kb
16,120 kb
16,130 kb
16,140 kb
16,150 kb
16,160 kb
16,170 kb
16,180 kb
sudden read depth change
split reads and large insert size

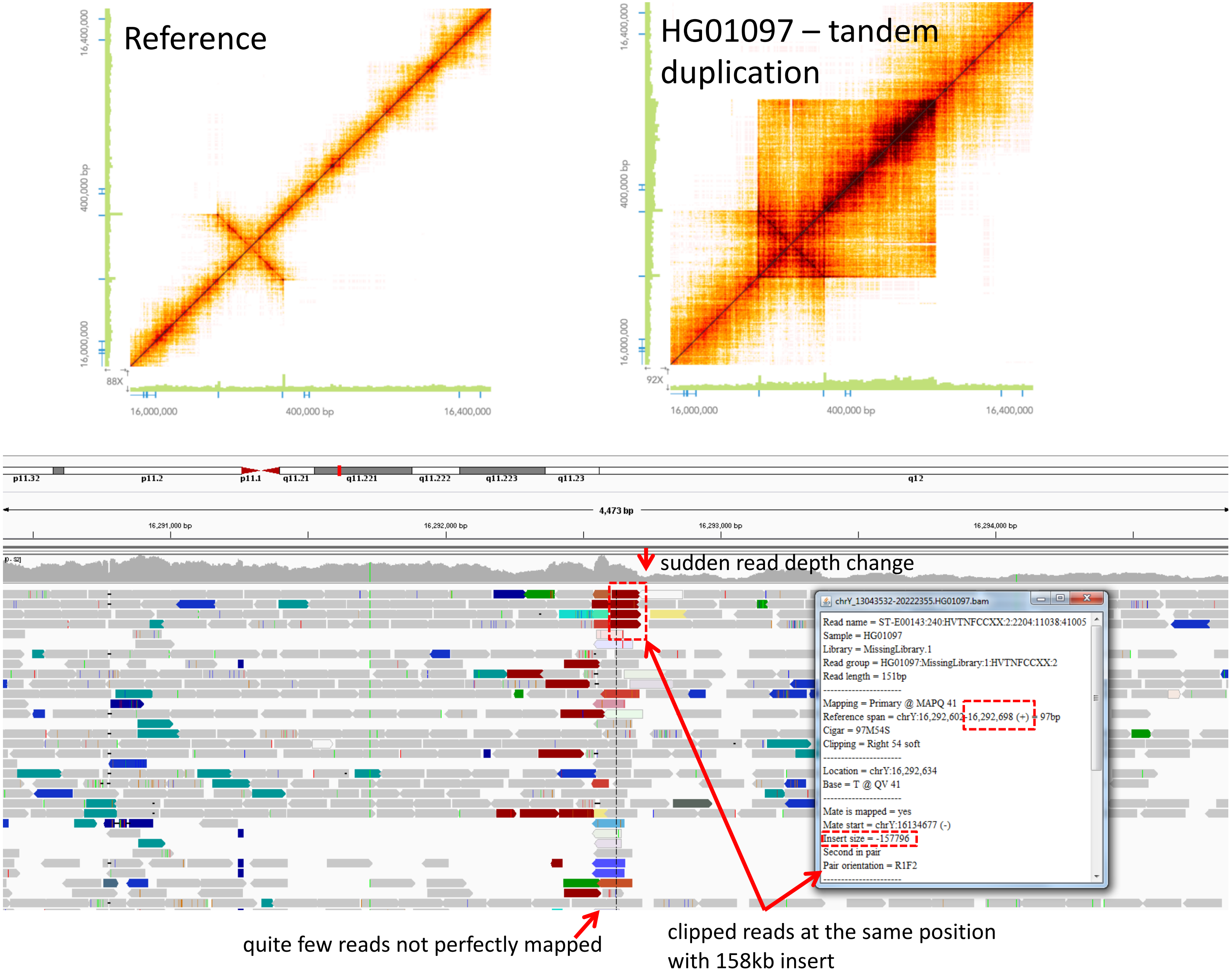
Reference
HG01097 – tandem duplication
16,000,000
400,000 bp
16,400,000
88X
92X
p11.32
p11.2
p11.1
q11.21
q11.221
q11.222
q11.223
q11.23
q12
4,473 bp
16,291,000 bp
16,292,000 bp
16,293,000 bp
16,294,000 bp
sudden read depth change
chrY_13043532-20222355.HG01097.bam
Read name = ST-E00143:240:HVTNFCCXX:2:2204:11038:41005
Sample = HG01097
Library = MissingLibrary.1
Read group = HG01097.MissingLibrary.1.HVTNFCCXX.2
Read length = 151bp
Mapping = Primary @ MAPQ 41
Reference span = chrY:16,292,602-16,292,698 (+) = 97bp
Cigar = 97M54S
Clipping = Right 54 soft
Location = chrY:16,292,634
Base = T @ QV 41
Mate is mapped = yes
Mate start = chrY:16134677 (-)
Insert size = -157796
Second in pair
Pair orientation = R1F2
quite few reads not perfectly mapped
clipped reads at the same position
with 158kb insert

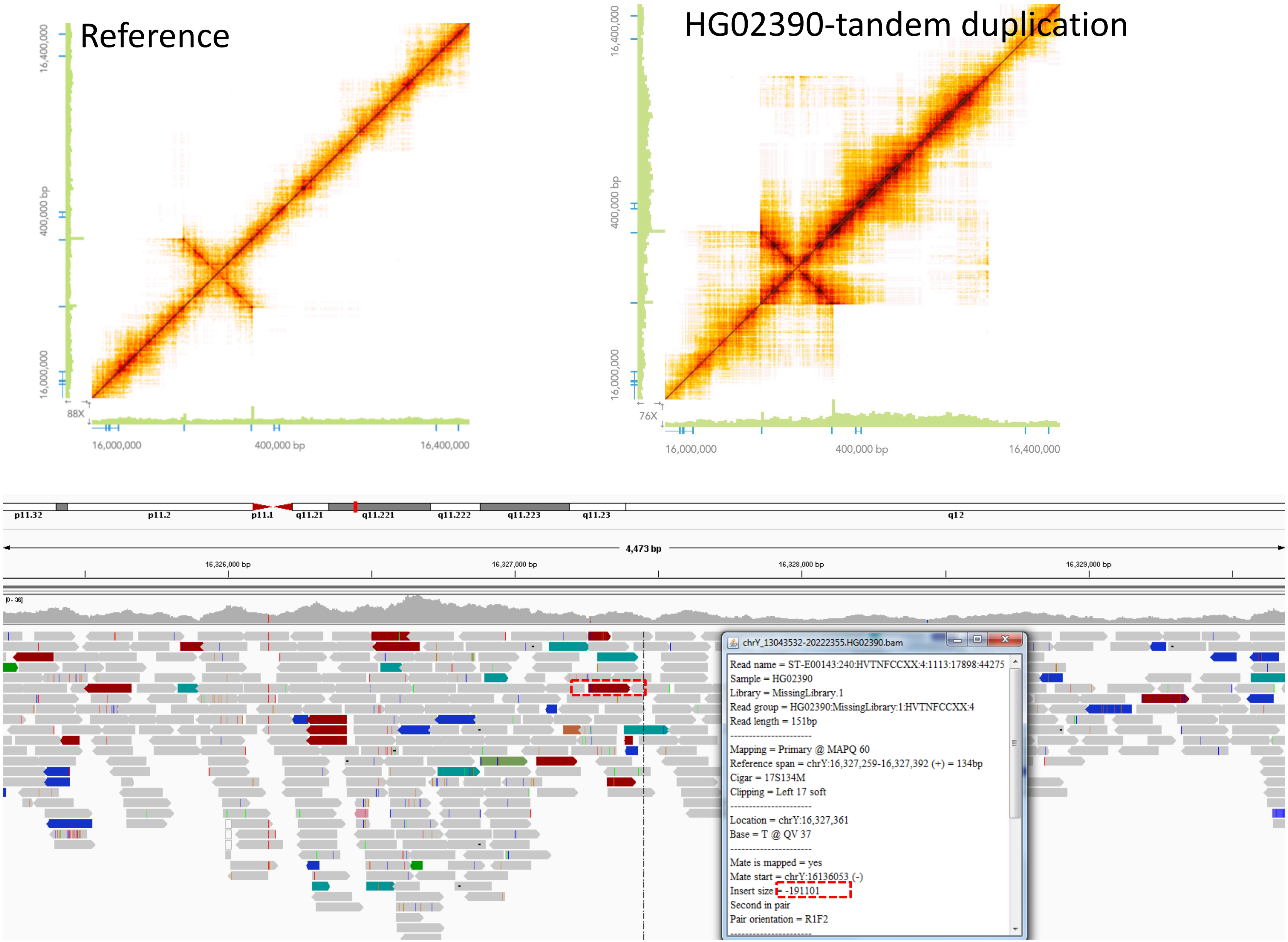

Too many SNPs in this region

A single informative read indicating an insert of 191 kb

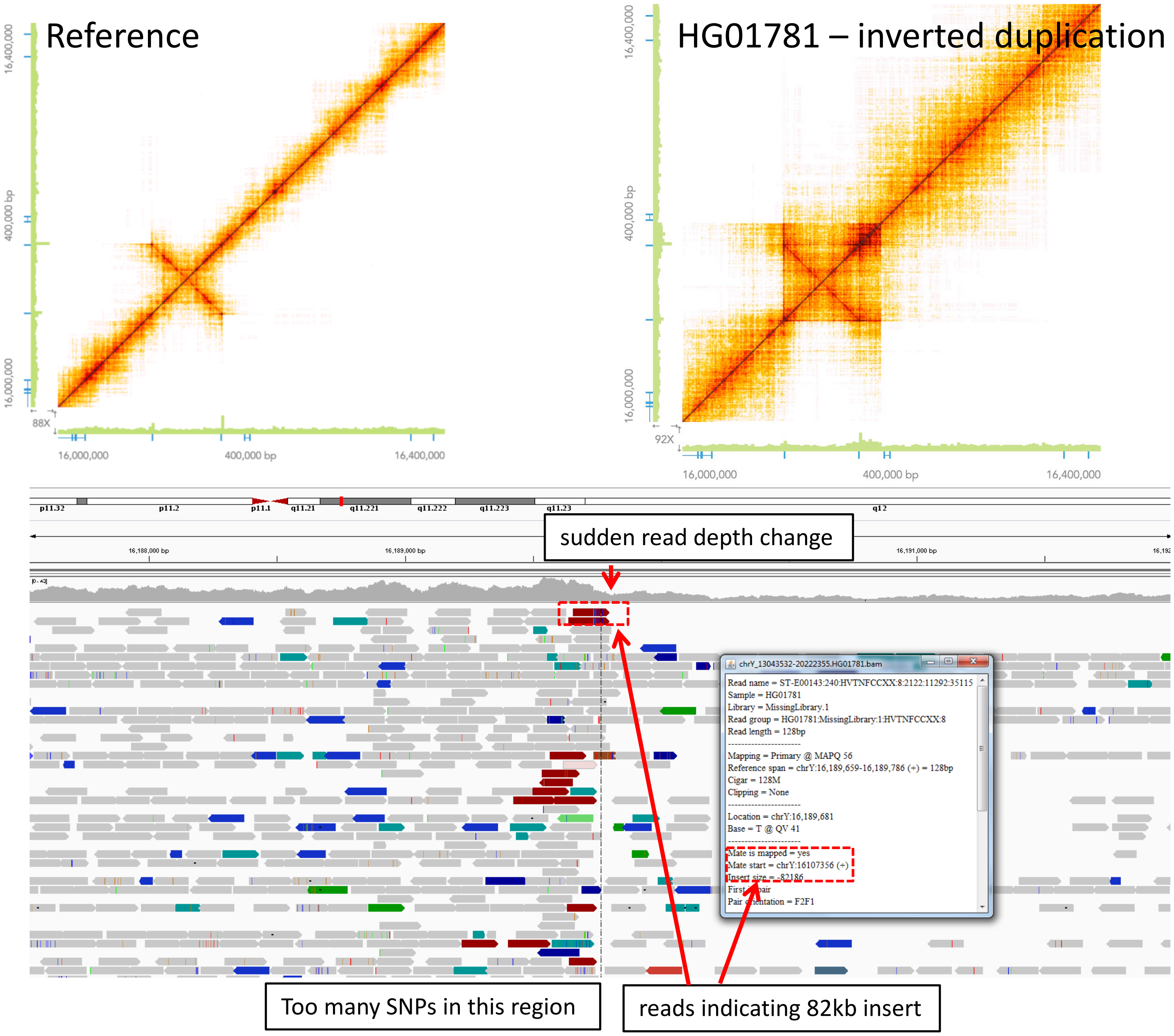
Reference
HG01781 – inverted duplication
16,000,000
400,000 bp
16,400,000
88X
92X
p11.32
p11.2
p11.1
q11.21
q11.221
q11.222
q11.223
q11.23
q12
16,188,000 bp
16,189,000 bp
16,191,000 bp
sudden read depth change
chrY_13043532-20222355.HG01781.bam
Read name = ST-E00143:240:HVTNFCCXX:8:2122:11292:35115
Sample = HG01781
Library = MissingLibrary.1
Read group = HG01781:MissingLibrary:1:HVTNFCCXX:8
Read length = 128bp
Mapping = Primary @ MAPQ 56
Reference span = chrY:16,189,659-16,189,786 (+) = 128bp
Cigar = 128M
Clipping = None
Location = chrY:16,189,681
Base = T @ QV 41
Mate is mapped = yes
Mate start = chrY:16107356 (+)
Insert size = -82186
Pair orientation = F2F1
Too many SNPs in this region
reads indicating 82kb insert

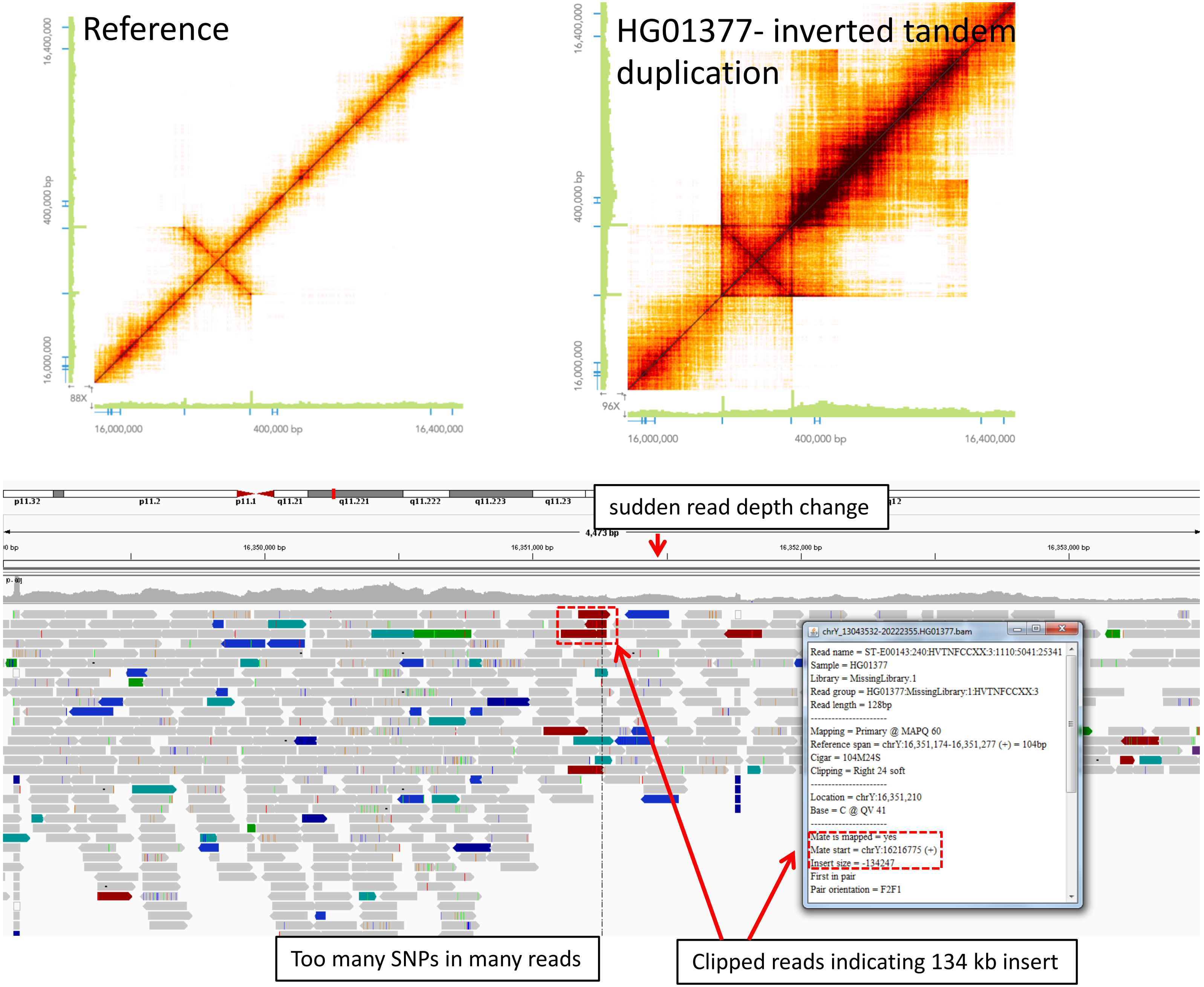
Reference
16,400,000
400,000 bp
16,000,000
88X
HG01377- inverted tandem duplication
96X
sudden read depth change
p11.32
p11.2
p11.1
q11.21
q11.221
q11.222
q11.223
q11.23
4,473 bp
16,350,000 bp
16,351,000 bp
16,352,000 bp
16,353,000 bp
chrY_13043532-20222355.HG01377.bam
Read name = ST-E00143:240:HVTNFCCXX:3:1110:5041:25341
Sample = HG01377
Library = MissingLibrary.1
Read group = HG01377:MissingLibrary:1:HVTNFCCXX:3
Read length = 128bp
Mapping = Primary @ MAPQ 60
Reference span = chrY:16,351,174-16,351,277 (+) = 104bp
Cigar = 104M24S
Clipping = Right 24 soft
Location = chrY:16,351,210
Base = C @ QV 41
Mate is mapped = yes
Mate start = chrY:16216775 (+)
Insert size = -134247
First in pair
Pair orientation = F2F1
Too many SNPs in many reads
Clipped reads indicating 134 kb insert

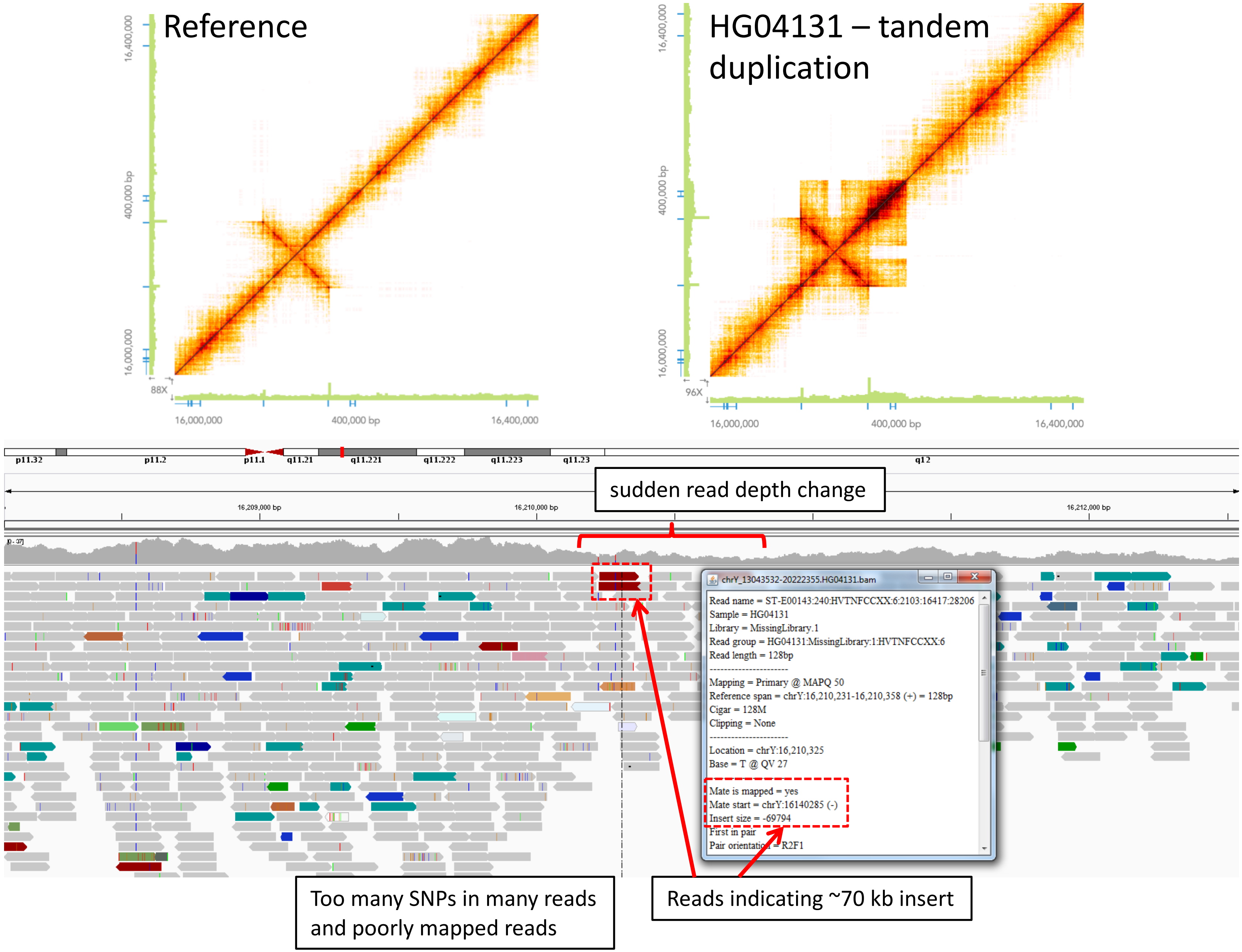
Reference
HG04131 – tandem duplication
16,000,000
400,000 bp
16,400,000
88X
96X
p11.32
p11.2
p11.1
q11.21
q11.221
q11.222
q11.223
q11.23
q12
16,209,000 bp
16,210,000 bp
16,212,000 bp
sudden read depth change
chrY_13043532-20222355.HG04131.bam
Read name = ST-E00143:240:HVTNFCCXX:6:2103:16417:28206
Sample = HG04131
Library = MissingLibrary.1
Read group = HG04131:MissingLibrary:1:HVTNFCCXX:6
Read length = 128bp
Mapping = Primary @ MAPQ 50
Reference span = chrY:16,210,231-16,210,358 (+) = 128bp
Cigar = 128M
Clipping = None
Location = chrY:16,210,325
Base = T @ QV 27
Mate is mapped = yes
Mate start = chrY:16140285 (-)
Insert size = -69794
First in pair
Pair orientation = R2F1
Too many SNPs in many reads and poorly mapped reads
Reads indicating ~70 kb insert

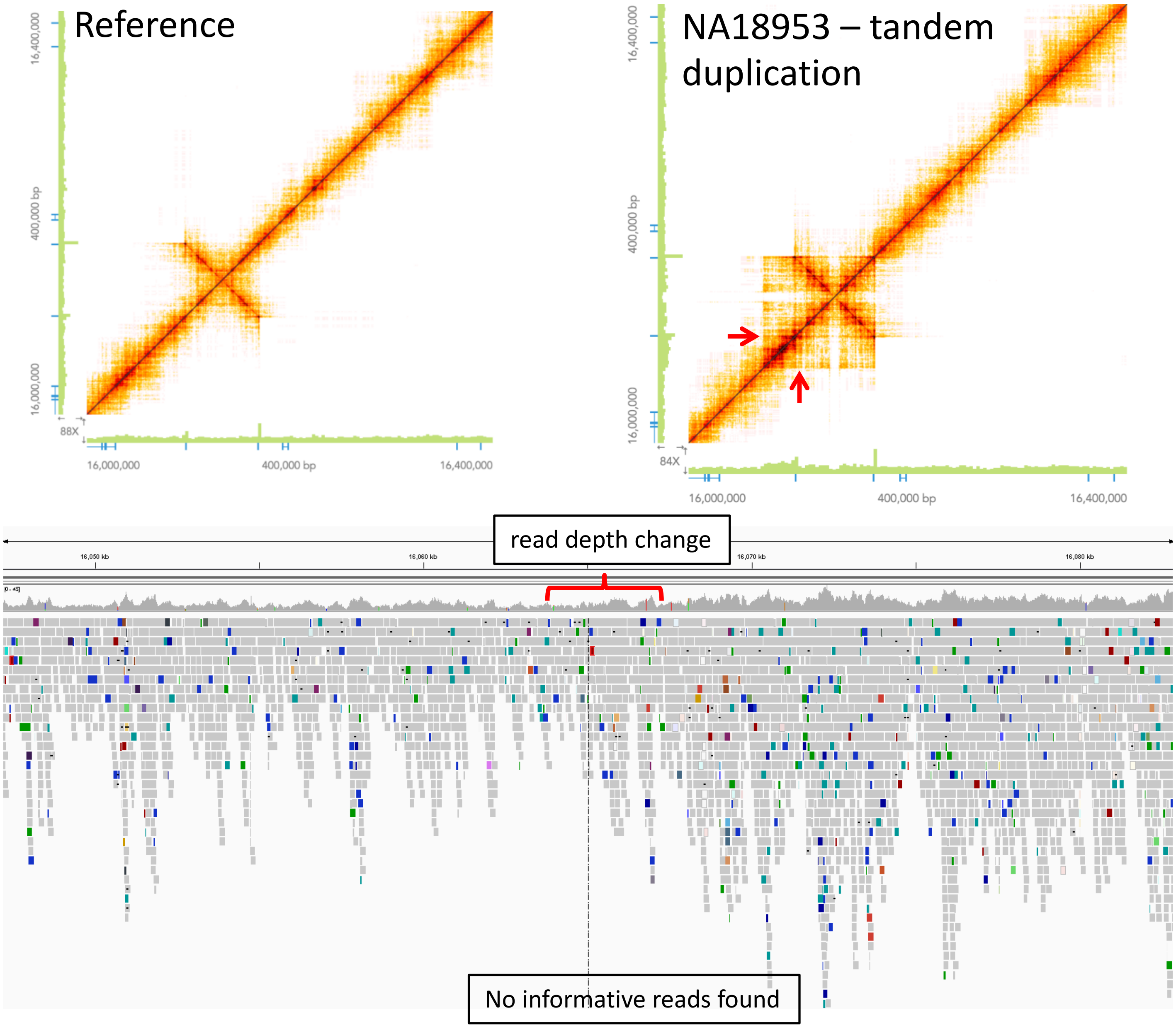
Reference
NA18953 – tandem duplication
16,000,000
400,000 bp
16,400,000
88X
84X
read depth change
16,050 kb
16,060 kb
16,070 kb
16,080 kb
No informative reads found

NA18953 – tandem duplication

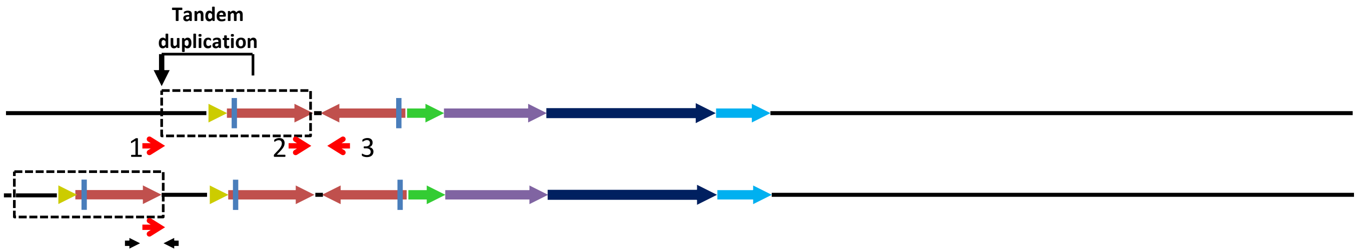

1st red arrow
Y:16,064,609 - 16,069,743 (5,134bp)
Break point should be in 1st red arrow according 10x and FISH result. Four left and right breakpoint primers within this region were designed. After PCR, it was narrowed down to 3 kb region with primer pair L3+LR (right). Sanger sequencing was used to sequence this product to identify the break point.

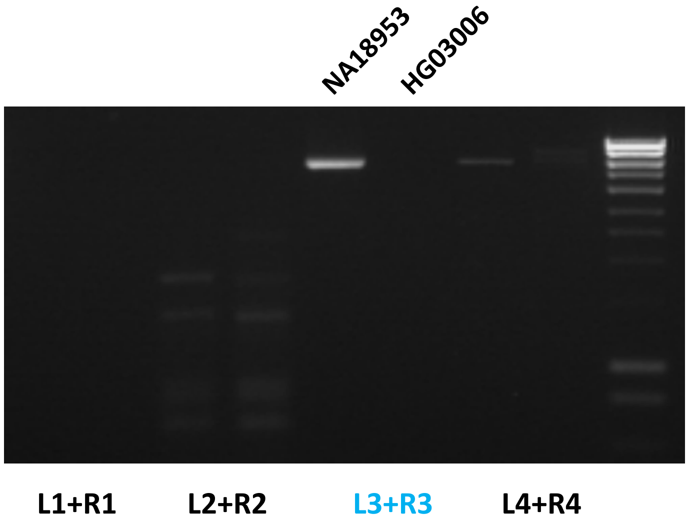

Four pairs of breakpoint PCR primers were tested on NA18953 and HG03006 (representing the reference sequence). Only pair L3+R3 generated a product.

2nd red arrow
Y: 16,125,186 - 16,130,314 (5,128bp)

3rd red arrow
Y: 16,136,175 - 16,141,303 (5,128bp)
