# Supplementary material for: Birth, expansion, and death of VCY-containing palindromes on the human Y chromosome
Source: Genome Biol. 2019 Oct 14;20:207. doi: 10.1186/s13059-019-1816-y (PMC6790999; doi:10.1186/s13059-019-1816-y)
Supplement: Supplementary file 4 — Additional file 4: Review history. (DOCX 19 kb) [file 13059_2019_1816_MOESM4_ESM.docx]

Review History

**First round of review**

**Reviewer 1**

**Are you able to assess all statistics in the manuscript, including the appropriateness of statistical tests used?**

There is no statistics

**Comments to author:**

Although descriptive, this is an interesting short paper, both for the approaches used to disentangle the complex changes that appear to have occurred in the palindrome region these authors studied. 

As mentioned below, the Conclusions section should probably be deleted, unless there really is something that can be learned from the results. I agree with the authors' suggestion that one would wish to understand how these sequence regions in non-recombining parts of the Y chromosome can evolve, as biologists tend (I believe) to stereotype non-recombining genome regions as lacking variability. This study sheds some light on processed that do generate variability in such a context, and I would have liked to see a brief review of what (if anything) is already known about such genome regions. This would be a valuable addition to this story, and would flesh out the currently very brief first section of the manuscript.


The English needs some revisions, and some further explanations would be helpful. 

1.     The term "locations", which first appears in line 92, is unclear. It seems not to mean the position in the phylogeny, or a geographic location (or at least Table 1, which is referred to in that line, doesn't include geographic locations), so I think that a different word is probably intended.

2.     The terminology should be explained, including names like HG00096. 

3.     The meaning of "shows no P8 copy number variation" is probably simply that the palindrome in question is the same as in the human reference genome. If so, this should be stated more clearly. If not, the meaning should still be expressed clearly, as it makes no sense that one instance of the palindrome shows no copy number variation, since, by definition, it is one single instance.

4.     Words like "decrease" should be accompanied by some information about what this is being compared with (i.e. decreased in comparsison with, or lower than, what?). In line 116, I think that the authors mean that the palindrome being discussed has fewer VCY copies than the one discussed previously. Such sentences should be made explicit.

5.     The tenses are sometimes confused (e.g. "lay" in the past tense, when the meaning is sometimes "lie" in the present tense). In general, the tenses should be checked by a native English speaker.

6.     In line 175, it should probably read "an increased read depth", and in line 204, it would be helpful to write "Three junctions…."

7.     The main paragraph on p. 12 is difficult to understand. I think that the meaning is 

The 12 samples with VCY copy numbers OTHER THAN two were CARRIED ON eight different Y haplogroups, dispersed throughout the SNP-based phylogeny constructed for the same set of Y chromosomes (Figure 5). Six samples each correspond to a single haplogroup, while the remaining six fell into two haplogroups, two in Y haplogroup J2a-M410 and four in O2-K18. THE breakpoint-PCRs DESCRIBED in the previous sections showed that all samples within THESE TWO groups shared the same breakpoint, AND The branch lengths IN THE TREE indicate that EACH OF these two haplogroups HAD a recent common ancestor, ~12,400 or 1,500 years ago. There were THUS eight distinct mutational events, at least two of WHICH were able to expand in the population, demonstrating that their carriers are fertile.

8.     The first sentence of the Discussion is an unnecessary repeat, as is most ot the Conclusions section.

9.     Line 232 should read "all with expression reported exclusively in male germ cells"

10.     In relation to the divergence values mentioned on page 11, the type of sites analysed should be explained. Were these synonymous sites in coding regions, or what?

11.     A comma is needed after the first word in line 247.

12.     The sentence in lines 254-5 could be easier to understand as "…. rearrangements are particularly common and account for most well-studied copy number variants found in Ychromosomes", and the next sentence would be clearer without the phrare about being ascertained for P8 alone (maybe say "despite being confined to just the P8 palindrome").

13.     In line 272, it should read "likely to have arisen"

14.     In line 287, the authors claim selective neutrality for the variants, but the observation that two variant types did not confer male sterility is not enough evidence that they are selectively neutral, and the claim should be made much more cautious. As this has already been made clear in the results section, and most of the Conclusions section merely repeats what has already been made clear, the entire section should probably be deleted.

**Reviewer 2**

**Are you able to assess all statistics in the manuscript, including the appropriateness of statistical tests used?**

There is no statistics

**Comments to author:**

In the manuscript "Birth, expansion and death of VCY-containing palindromes on the human Y chromosome" Shi and colleagues, using a variety of innovative technical approaches, report the cytogenetic and molecular characterization of eight different structural variations involving the Y chromosome palindrome P8, including the birth of a new large palindrome. Based on the observed sequence changes, the Authors hypothesize different molecular mechanisms for the mutational events. To my knowledge, this is the first detailed report on the cytogenetic mutability of a large palindromic sequence, which is fundamental to understand how these structures, commonly present on sex chromosomes, evolve.
The manuscript is well written and extremely clear and the Authors' conclusions are well supported by the experimental results. 
I only have a few minor comments:
- Six out of eight mutations occur in terminal branches of the phylogenetic tree, while the other two also occur in fairly recent sub-terminal branches. No ancient mutations have been observed. I wonder if this result can be the consequence of negative selection against copy number variation of the VCY gene.
- DNAs analyzed in the 1,000 genomes project come from immortalized cells, which are prone to cytogenetic rearrangements. For the six mutations that have been observed in a single male, the possibility of somatic mutations rather than germ-line mutations cannot be excluded. 
- The Authors recognize that VCY genes are members of a gene family that also contains X-chromosome members designated VCX (page 10, lines 231-232). However, VCX genes are completely ignored in the rest of the discussion. VCX and VCY genes are characterized by high sequence homology due to X-Y gene conversion, which is a mechanism of DSB repair and thus in some way relevant for the present manuscript. Also VCX and VCY genes have probably the same expression pattern (based on the references cited in the manuscript) therefore, considerations about "tolerance of different copy numbers..." (page 11 lines 247-249) should also take into consideration the contribution of VCX genes.
- Page 4, line 90-91: the sentence "these were confirmed by...CGH data" seems redundant with respect to the sentence reported two lines above ("increase or decrease...CGH intensity"). 
-Page 9, line 204: Please, check the sentence "Three (HG02390, HG01377 and)"

**Authors Response**

**Point-by-point responses to the reviewers’ comments:**

Comment

*Response*

Although descriptive, this is an interesting short paper, both for the approaches used to disentangle the complex changes that appear to have occurred in the palindrome region these authors studied. 

As mentioned below, the Conclusions section should probably be deleted, unless there really is something that can be learned from the results. I agree with the authors' suggestion that one would wish to understand how these sequence regions in non-recombining parts of the Y chromosome can evolve, as biologists tend (I believe) to stereotype non-recombining genome regions as lacking variability. This study sheds some light on processed that do generate variability in such a context, and I would have liked to see a brief review of what (if anything) is already known about such genome regions. This would be a valuable addition to this story, and would flesh out the currently very brief first section of the manuscript. 

*Response: A Conclusions section is part of the Genome Biology article format, so we have retained this, but taken more care to avoid unnecessary repetition. We have added more material on the structural variation of the Y chromosome to create an additional paragraph in the Background section, as suggested.* 


The English needs some revisions, and some further explanations would be helpful. 

1. The term "locations", which first appears in line 92, is unclear. It seems not to mean the position in the phylogeny, or a geographic location (or at least Table 1, which is referred to in that line, doesn't include geographic locations), so I think that a different word is probably intended. 

*Response: We mean the position in the phylogeny, and have changed the term to “branches”, here and in line 97.* 

2. The terminology should be explained, including names like HG00096. 

*Response: This is the name of a sample, as used by the 1000 Genomes Project, and initially assigned by the Coriell Institute for Medical Research which distributes the samples. We have now explained this briefly in the text where the first sample name occurs.* 

3. The meaning of "shows no P8 copy number variation" is probably simply that the palindrome in question is the same as in the human reference genome. If so, this should be stated more clearly. If not, the meaning should still be expressed clearly, as it makes no sense that one instance of the palindrome shows no copy number variation, since, by definition, it is one single instance. 

*Response: The reviewer’s interpretation is correct, and we have re-phrased this as “which matches the reference sequence P8 copy number”.* 

4. Words like "decrease" should be accompanied by some information about what this is being compared with (i.e. decreased in comparsison with, or lower than, what?). In line 116, I think that the authors mean that the palindrome being discussed has fewer VCY copies than the one discussed previously. Such sentences should be made explicit. 

*Response: The reviewer’s reading is again correct, and we have made the comparison more explicit, here and throughout.* 

5. The tenses are sometimes confused (e.g. "lay" in the past tense, when the meaning is sometimes "lie" in the present tense). In general, the tenses should be checked by a native English speaker. 

*Response: The tenses have been checked by a native English speaker throughout.* 

6. In line 175, it should probably read "an increased read depth", and in line 204, it would be helpful to write "Three junctions…." 

*Response: Both changes have been made as suggested.* 

7. The main paragraph on p. 12 is difficult to understand. I think that the meaning is 

The 12 samples with VCY copy numbers OTHER THAN two were CARRIED ON eight different Y haplogroups, dispersed throughout the SNP-based phylogeny constructed for the same set of Y chromosomes (Figure 5). Six samples each correspond to a single haplogroup, while the remaining six fell into two haplogroups, two in Y haplogroup J2a-M410 and four in O2-K18. THE breakpoint-PCRs DESCRIBED in the previous sections showed that all samples within THESE TWO groups shared the same breakpoint, AND The branch lengths IN THE TREE indicate that EACH OF these two haplogroups HAD a recent common ancestor, ~12,400 or 1,500 years ago. There were THUS eight distinct mutational events, at least two of WHICH were able to expand in the population, demonstrating that their carriers are fertile. 

*Response: We thank the reviewer for these careful suggestions, and have incorporated them.* 

8. The first sentence of the Discussion is an unnecessary repeat, as is most ot the Conclusions section. 

*Response: We have modified the first sentence of the Discussion. As explained earlier, we have retained the Conclusions section.* 

9. Line 232 should read "all with expression reported exclusively in male germ cells" 

*Response: Changed as suggested.* 

10. In relation to the divergence values mentioned on page 11, the type of sites analysed should be explained. Were these synonymous sites in coding regions, or what? 

*Response: The 4.3% divergence between human and chimpanzee is the average over all nucleotides in the coding region, and this has been clarified in the text.* 

11. A comma is needed after the first word in line 247. 

*Response: We have added this comma.* 

12. The sentence in lines 254-5 could be easier to understand as "…. rearrangements are particularly common and account for most well-studied copy number variants found in Ychromosomes", and the next sentence would be clearer without the phrare about being ascertained for P8 alone (maybe say "despite being confined to just the P8 palindrome"). 

*Response: We have modified the first sentence in the way suggested. However, we do not agree with the suggested change to the following sentence, since some of the variants affect regions of the genome outside the P8 palindrome (a 78.8 kb region, as described in the Background).* 

13. In line 272, it should read "likely to have arisen" 

*Response: Changed as suggested.* 

14. In line 287, the authors claim selective neutrality for the variants, but the observation that two variant types did not confer male sterility is not enough evidence that they are selectively neutral, and the claim should be made much more cautious. As this has already been made clear in the results section, and most of the Conclusions section merely repeats what has already been made clear, the entire section should probably be deleted. 

*Response: As described in the response to Reviewer 2, we have carried out additional analyses to investigate the neutrality of the P8 variants. While these support neutrality, we agree with Reviewer 1 that this claim should be made cautiously, and have rephrased it as “These variants therefore do not have strongly deleterious consequences and are consistent with evolutionary neutrality, and ….”. Our response about the Conclusions section has been given above.* 


Reviewer #2: In the manuscript "Birth, expansion and death of VCY-containing palindromes on the human Y chromosome" Shi and colleagues, using a variety of innovative technical approaches, report the cytogenetic and molecular characterization of eight different structural variations involving the Y chromosome palindrome P8, including the birth of a new large palindrome. Based on the observed sequence changes, the Authors hypothesize different molecular mechanisms for the mutational events. To my knowledge, this is the first detailed report on the cytogenetic mutability of a large palindromic sequence, which is fundamental to understand how these structures, commonly present on sex chromosomes, evolve. 
The manuscript is well written and extremely clear and the Authors' conclusions are well supported by the experimental results. 

*Response: We thank the reviewer for these comments.* 

I only have a few minor comments: 
- Six out of eight mutations occur in terminal branches of the phylogenetic tree, while the other two also occur in fairly recent sub-terminal branches. No ancient mutations have been observed. I wonder if this result can be the consequence of negative selection against copy number variation of the VCY gene. 

*Response: This is an interesting suggestion. It is not easy to test, not least because of the small numbers of mutations, but we can compare the frequencies of P8 variants with synonymous variants (assumed to be neutral) and non-synonymous variants (assumed to be on average subject to weak negative selection) in the same dataset. The results of such a test (Chi square with two degrees of freedom) were: 

synonymous vs non-synonymous: Pearson: 14.92 (p= 0.00058) 
VCY vs synonymous: Pearson: 1.002 (p= 0.60583) 
VCY vs non-synonymous: Pearson: 7.351 (p= 0.02534) 

These findings show that the frequency distributions of synonymous and non-synonymous variants are significantly different, as found in many previous studies, and interpreted as the consequence of weak negative selection against many of the non-synonymous variants. The VCY distribution is significantly different from the non-synonymous distribution, but not different from the synonymous one. This finding supports the idea of neutrality for the VCY structural variants. We have added this analysis to the text as an additional paragraph at the end of the Results section.* 

- DNAs analyzed in the 1,000 genomes project come from immortalized cells, which are prone to cytogenetic rearrangements. For the six mutations that have been observed in a single male, the possibility of somatic mutations rather than germ-line mutations cannot be excluded. 

*Response: We agree with the reviewer that this is a possibility that cannot be entirely excluded. However, somatic mutations would initially be mosaic, and there was no evidence for mosaicism in any of the P8 variants. We have therefore added a paragraph on this point at the beginning of the Discussion.* 

- The Authors recognize that VCY genes are members of a gene family that also contains X-chromosome members designated VCX (page 10, lines 231-232). However, VCX genes are completely ignored in the rest of the discussion. VCX and VCY genes are characterized by high sequence homology due to X-Y gene conversion, which is a mechanism of DSB repair and thus in some way relevant for the present manuscript. Also VCX and VCY genes have probably the same expression pattern (based on the references cited in the manuscript) therefore, considerations about "tolerance of different copy numbers..." (page 11 lines 247-249) should also take into consideration the contribution of VCX genes. 

*Response: This is a good point. We have added another reference to the VCX gene cluster in this paragraph.* 

- Page 4, line 90-91: the sentence "these were confirmed by...CGH data" seems redundant with respect to the sentence reported two lines above ("increase or decrease...CGH intensity"). 

*Response: We can see that this may seem redundant, but experience has shown us that it is still wise to re-confirm findings from large-scale projects that have passed the project’s QC before investing a lot of time and effort in studying them further, and it seemed worth emphasising this at the expense of some redundancy. We have changed “confirmed” to “re-confirmed”.* 

-Page 9, line 204: Please, check the sentence "Three (HG02390, HG01377 and)" 

*Response: We thank the reviewer for spotting this, and have inserted the missing name (HG01097).*
